# Expression and function of ATP-dependent potassium channels in zebrafish islet β-cells

**DOI:** 10.1098/rsos.160808

**Published:** 2017-02-08

**Authors:** Christopher H. Emfinger, Alecia Welscher, Zihan Yan, Yixi Wang, Hannah Conway, Jennifer B. Moss, Larry G. Moss, Maria S. Remedi, Colin G. Nichols

**Affiliations:** 1 Department of Cell Biology and Physiology, Washington University in St Louis, St Louis, MO, USA; 2 Division of Endocrinology, Metabolism, and Lipid Research, Department of Medicine, Washington University in St Louis, St Louis, MO, USA; 3 Center for the Investigation of Membrane Excitability Diseases, Washington University in St Louis, St Louis, MO, USA; 4 Division of Endocrinology, Metabolism, and Nutrition and DMPI, Duke University Medical Center, Durham, NC, USA

**Keywords:** K_ATP_, metabolism, pancreas, zebrafish, ion channels

## Abstract

ATP-sensitive potassium channels (K_ATP_ channels) are critical nutrient sensors in many mammalian tissues. In the pancreas, K_ATP_ channels are essential for coupling glucose metabolism to insulin secretion. While orthologous genes for many components of metabolism–secretion coupling in mammals are present in lower vertebrates, their expression, functionality and ultimate impact on body glucose homeostasis are unclear. In this paper, we demonstrate that zebrafish islet β-cells express functional K_ATP_ channels of similar subunit composition, structure and metabolic sensitivity to their mammalian counterparts. We further show that pharmacological activation of native zebrafish K_ATP_ using diazoxide, a specific K_ATP_ channel opener, is sufficient to disturb glucose tolerance in adult zebrafish. That β-cell K_ATP_ channel expression and function are conserved between zebrafish and mammals illustrates the evolutionary conservation of islet metabolic sensing from fish to humans, and lends relevance to the use of zebrafish to model islet glucose sensing and diseases of membrane excitability such as neonatal diabetes.

## 1. Introduction

In the pancreatic β-cell, ATP-sensitive potassium (K_ATP_) channels link glucose metabolism and insulin secretion and are essential to the normal regulation of plasma glucose and other nutrients [1,2]. At low glucose, intracellular [ATP]/[ADP] is low, and K_ATP_ channels are open, hyperpolarizing the cell membrane. As plasma glucose rises, it enters β-cells through glucose transporter 2 (GLUT2), increasing [ATP]/[ADP] which closes K_ATP_ channels, inducing plasma membrane depolarization and opening voltage-dependent Ca^2+^ channels (VDCCs). Calcium influx through VDCCs subsequently triggers insulin secretion (electronic supplementary material, figure S1*a*). The predominant role of K_ATP_ channels is illustrated by the striking disease consequences of K_ATP_ mutations. Loss-of-function mutations result in congenital hyperinsulinism [3], whereas gain-of-function (GOF) mutations cause neonatal diabetes mellitus (NDM) [4,5], and polymorphisms are associated with the development of type 2 diabetes [6].

K_ATP_ channels have been well characterized in multiple mammalian tissues, and mechanisms coupling metabolism to insulin secretion have been well established in humans and other mammals. However, whether K_ATP_ channel structure or function, as well as insulin secretion mechanisms, are conserved in and physiologically significant for lower vertebrates remains unclear. Studies in the zebrafish, *Danio rerio*, indicate that K_ATP_ channels may be physiologically significant in fishes: treatment of larvae with pharmacological activators of mammalian K_ATP_ channels or transgenic expression of mammalian K_ATP_ channels with GOF mutations is sufficient to raise larval whole-body glucose [7]. Conversely, treatment of larvae with compounds that can close mammalian K_ATP_ channels, or transgenic expression of mammalian K_ATP_ channels with dominant-negative mutations, is sufficient to lower whole-larval glucose [7,8].

However, there have been very few mechanistic studies of insulin secretion in fishes, only a handful of papers even mention K_ATP_ channels in zebrafish [7,9–11], and direct analysis of K_ATP_ expression and functional characterization is lacking. We have now developed approaches for efficiently identifying and isolating zebrafish islets, and for electrophysiological analysis of isolated β-cells. We show that zebrafish β-cells express functional K_ATP_ channels with similar regulation, subunit composition and pharmacology to their mammalian counterparts, and that pharmacologic K_ATP_ channel openers can disrupt glucose tolerance in adult fish. Our results indicate that K_ATP_ channels serve a highly conserved role in regulating metabolism in zebrafish, and that zebrafish may function as valuable models for metabolic studies.

## 2. Material and methods

### 2.1. Nucleotide and amino acid alignments and identity determination

Comparisons of nucleotide and amino acid sequences of the orthologues of mammalian K_ATP_ channel components were completed in DNASTAR Lasergene MegAlign using ClustalW alignment. Search query IDs giving the sequences analysed for the components studied are indicated in the electronic supplementary material, table S1 for the nucleotide alignments and electronic supplementary material, table S2 for amino acid alignments. Amino acid sequences were analysed using InterPro v. 5 [12] (https://www.ebi.ac.uk/interpro/) with Phobius [13] to determine predicted transmembrane, cytoplasmic and extracellular residues.

### 2.2. Animal lines and maintenance

Transparent Casper zebrafish [14] were used for injection experiments. Zebrafish expressing eGFP under the insulin promoter (Tg(−1.0ins:eGFP)sc1) were used for islet isolation studies [15]. These fish were crossed into the Casper background for three generations (until external pigmentation was lost) and were maintained in the Washington University zebrafish facility. Details of standard operating procedures for the facility can be found at http://zebrafishfacility.wustl.edu/documents.html. All procedures were approved by the Washington University in St Louis IACUC.

### 2.3. Islet and β-cell isolation

Zebrafish islets were isolated as described previously [15], with minor modifications. Briefly, fish were euthanized using cold-shock (8°C water immersion) followed by decapitation. Fish were rolled onto their right sides and the exterior skin, and scales were removed using surgical forceps to expose the abdomen. Visceral organs were removed by gently applying pressure using forceps until fully separated. The islets were identified at the intersection of hepatic and bile ducts with the intestine (located using the gall bladder and spleen as regional indicators) and confirmed by eGFP fluorescence. Islets were removed by gently pinching ducts with forceps and separating the islets from the surrounding tissues.

Exocrine tissues surrounding islets were digested with collagenase (Sigma C9263, 0.4 mg ml^−1^ in Hank's buffered salt solution, 0.5 ml/5–10 islets), during incubation at 29°C for 20 min, shaking gently every 5 min. Islets were then placed in RPMI (ThermoFisher 11875-093) supplemented with 1 mM HEPES, antibiotic solution (Sigma A5955, 10 ml l^−1^ solution), 10% fetal bovine serum and diluted with glucose-free RPMI to final glucose concentration of 6.67 mM.

For experiments involving individual β-cells, islets were dispersed with StemPro Accutase (ThermoFisher A11105) for 10 min at 37°C and clumps of cells were incubated a second time in the same conditions for 2 min. Dispersed cells were washed with media and re-suspended in less than or equal to 100 µl of media, then transferred to glass shards cut from coverslips. Cells were allowed to adhere for 30 min in incubator (28°C, 0% CO_2_) on shards before being completely covered with media and incubated overnight in the same conditions.

### 2.4. Chemicals

Salts and glucose were purchased from Sigma Aldrich. Diazoxide (D9035), pinacidil (P154), tolbutamide (T0891) and glibenclamide (G0639) were purchased from Sigma Aldrich.

### 2.5. Whole-cell voltage-clamp and excised inside-out patch-clamp experiments

Whole-cell and inside-out excised-patch voltage-clamp experiments were performed as described for mammalian β-cells [16], with minor modifications. Isolated β-cells adhering to glass shards were transferred to bath solutions. Bath solution for whole-cell experiments was Tyrode's solution containing 137 mM NaCl, 5.4 mM KCl, 1 mM MgCl_2_, 2 mM CaCl_2_, 0.33 mM NaH_2_PO_4_, 5 mM HEPES and 1 mM glucose. Bath solution for inside-out excised patch experiments (K-INT) contained 140 mM KCl, 10 mM HEPES and 1 mM K-EGTA adjusted to pH 7.4 with KOH. In experiments testing, ADP or drug action on K_ATP_ channels, 0.5 mM free Mg^2+^ was added to K-INT except for high [ATP] lanes. The amount of MgCl_2_ used to reach this free Mg^2+^ concentration was calculated using the CaBuf program (no longer accessible at webpages previously cited in other articles (ftp://ftp.cc.kuleuven.ac.be/pub/droogmans/cabuf.zip, as well as other sources). We are happy to provide this to any requestors. For drug studies, drugs were kept as 100 mM stock solutions in DMSO (except diazoxide, which was kept at 300 mM in DMSO). Drug stocks were diluted to 100 µM in K-INT, with DMSO added to K-INT and ATP solutions to match the drug solution (0.1% DMSO).

Glass electrodes were pulled from Kimble-Chase 2502 micro-haematocrit capillary tubes using a P-97 puller (Sutter instruments) to yield 2–4 MΩ tips, when filled with K-INT. Recordings of currents were made using an Axopatch1B or Axopatch 200B amplifier and Axon pCLAMP software from Molecular Devices. For excised patches (seal greater than 1 GΩ), membrane potential was kept constant at +50 mV. Once lifted, the pipet was moved through a mineral oil gate [17] to rip the cell free, leaving the patch in the micropipette. Patches were sequentially exposed to varying concentrations of ATP or ATP and activators, as noted. For whole-cell recordings, membrane potential was held at −70 mV and repeatedly ramped between −120 and +40 mV.

### 2.6. RNA isolation, cDNA preparation and channel subunit PCR

As young adult zebrafish pancreata typically contain only one to three large islets (approx. 10 000 cells), biological replicates were designated as pools of islets from eight to 15 fish each. RNA was isolated from pooled islets using the QIAGEN RNEasy mini-kit. cDNA was synthesized from isolated RNA with the ThermoFisher High-Capacity cDNA reverse transcription kit. Genomic DNA was isolated from zebrafish hearts using the gMax mini-kit (IBI scientific). Primers for ion channel PCRs are listed in the electronic supplementary material, table S5. Primers were designed using the Primer-BLAST NCBI online tool and checked for specificity for the selected genes (http://www.ncbi.nlm.nih.gov/tools/primer-blast/) [18]. PCRs were run with Platinum Taq High-fidelity polymerase (ThermoFisher 11304–11) and products were separated on 1.5–2% agarose gels in TAE buffer and visualized with ChemiDoc. MP (Bio-Rad). PCRs on cDNAs from *n* > 5 separate pools of islets and *n* > 5 genomic DNAs were analysed, with figure 3*a* being a representative sample.

For cell sorting, islets were dispersed as above and sorted using a BD FACSAria II (BD Biosciences) at the Washington University Flow Cytometry and Fluorescence Activated Cell Sorting Core (http://pathology.wustl.edu/Research/cores/facs/index.php). RNA was extracted from sorted cells as described [19] using TRIzol (ThermoFisher 15596026) and chloroform (Sigma C0549). DNA was removed from RNA samples using DNAseI (ThermoFisher 18068015) for islet samples and TURBO DNA-free kit (ThermoFisher AM1907) for sorted cell samples prior to reverse transcription. The FACSAria II data file is included as online material.

### 2.7. Adult zebrafish injection studies

Injections were performed as previously described [20], with modifications. Adult Casper zebrafish of both sexes, approximately six to eight months of age, were anaesthetized by cold water immersion. Animals were then transferred to pre-weighed cold water-soaked sponges in Petri dishes with indentations cut to maintain hydration while holding fish immobilized. Fish were injected (10 µl gBW^−1^) intraperitoneally (IP) using disposable 32 G needles (Acuderm) with Luer-Lock hubs on gas-tight 50 µl syringes (Hamilton 1705). For the IP glucose tolerance test, all solutions were prepared in 20% DMSO in 1 × PBS with 5 mg ml^−1^ phenol red. Following injection, animals were returned to warm water (28°C) for recovery. For plasma glucose measurements at indicated time points, individual fish were euthanized by immersion in cold water followed by decapitation across the gills. OneTouch Ultra glucometers were used to measure blood glucose by placing a glucometer strip at sectioned heart at time of decapitation.

### 2.8. Data analyses

Initial experiments with ATP inhibition on excised patches from zebrafish established a variability similar to that seen for mammalian channels under similar conditions [21]. [ATP]–response relationships were fitted with a modified Hill equation:

2.1 $$I_{\mathrm{rel}}=\frac{1}{1+{\left([\text{ATP}]\text{/I}{\text{C}}_{50}\right)}^{n_{H}}}\;,$$

where *I*_rel_ is the current relative to that in zero ATP; IC_50_ is the ATP concentration at which channels are half-maximally inhibited; [ATP] is the concentration of ligand; and *n*_H_ is the Hill coefficient. Fitting was done with GraphPad Prism software, using least-squares variable-slope log([*inhibitor*]) versus normalized response function, with a resulting R-squared value of 0.97.

Statistical comparisons between the various datasets were performed in GraphPad Prism, and the specific tests are indicated in the relevant figure legends. Datasets were tested for normality (Shapiro–Wilk) and whether variances were statistically different between groups (Bartlett's test). Where normality and variance assumptions were met, ANOVA with Tukey's multiple comparisons tests (three or more groups) or Student's *t*-test with Welch's correction (two groups) was used. As some datasets showed non-normal data or significantly different variances within the groups, non-parametric tests were used (for tests of three or more groups, Kruskal–Wallis ranked test, with Dunn's multiple comparisons; Mann–Whitney for two-group analyses). To improve interpretability of glucose tolerance tests, the values were log-transformed before statistical analysis.

### 2.9. Online supplementary material

**Figure 1.**
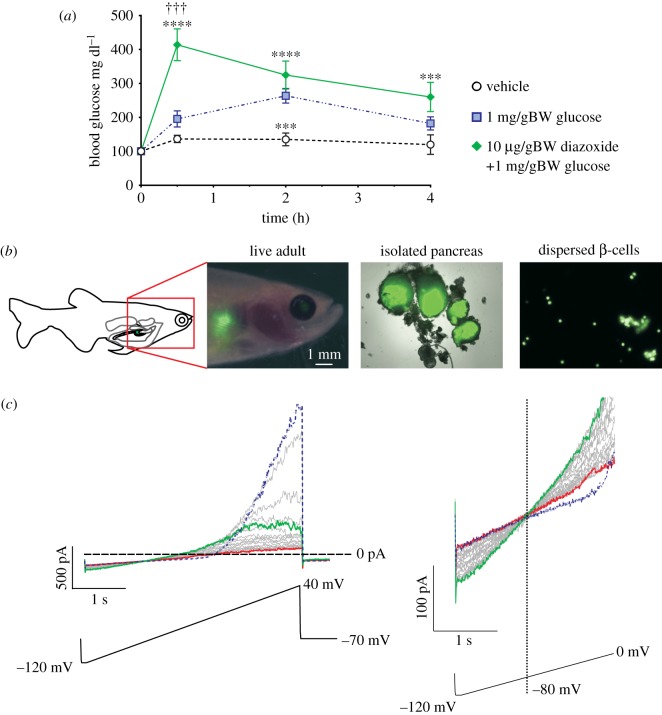
Whole-cell voltage-clamp of zebrafish β-cells reveals functional K_ATP_ channels. (*a*) Glucose tolerance in adult zebrafish. Blood glucose at each time point in (*a*) was compared between groups by ANOVA with Tukey's multiple comparisons on log-transformed datasets. *n* = 7–13 at each time point except for the baseline values which were 24. ^***^p < 0.001, group versus vehicle; ^****^p < 0.0001, group versus vehicle; *p*-values are from Tukey's multiple comparisons test following ANOVA of log-transformed values. ^†††^*p* < 0.001, group versus glucose, also from Tukey's multiple comparisons test. Data in this panel are compiled from multiple injection experiments performed over several days. (*b*) Expression of eGFP in the fish pancreas allows visualization of β-cells in live adults (left image), isolated islets (middle image) and dispersed β-cells (right image). Scale is indicated for the live adult image. The middle and right images are at 20× and 40×, respectively. For image panels, bright-field and fluorescence images were superimposed for adult fish and whole islets. Adult fish bright-field image was contrast-enhanced prior to superimposing it with the fluorescence image to enhance visibility in the final combined image. (*c*) Whole-cell voltage-clamp detection of K_ATP_ in zebrafish β-cells. Voltage ramps (lower) were applied from −120 to +40 mV over 4 s. Following break-in, the initial ramp (blue) elicits large voltage-dependent K currents above −30 mV. These currents gradually run down in successive voltage ramps, and a weakly inwardly rectifying K_ATP_ conductance gradually increases to maximal (green) and then in turn runs down to baseline (red). Right panel shows currents between −120 and 0 mV for more clear visualization of K_ATP_ currents.

Included online are three electronic supplementary material tables and six electronic supplementary material figures. Electronic supplementary material, table S1: references for nucleotide sequence alignments and identity determination. Electronic supplementary material, table S2: references for amino acid sequence alignments and identity determination. Electronic supplementary material, table S3: sequences and relevant data for the primers used in PCR reactions for the K_ATP_ channel subunits in zebrafish. Electronic supplementary material, figure S1: amino acid and nucleotide identities for K_ATP_ channel subunits in humans and zebrafish. Electronic supplementary material, figure S2: the alignment of zebrafish Kir6.x subunits relative to their mammalian orthologues highlighting residues of interest and functional domains. Electronic supplementary material, figure S3: alignment of zebrafish SURx subunit sequences relative to mammalian sequences, highlighting residues of putative functional significance in mammalian subunits that are or are not conserved in zebrafish orthologues. Electronic supplementary material, figure S4: uncut images of gels for PCR of K_ATP_ channel subunit components in islets (cf. figure 3). Electronic supplementary material, figure S5: uncut images of gels for PCR of K_ATP_ channel subunits in sorted β-cells (cf. figure 3). Electronic supplementary material, figure S6: unmodified traces for tolbutamide and glibenclamide (cf. figure 4). Electronic supplementary material, figure S7: unmodified bright-field and fluorescence images used for the composite images in figure 1.

## 3. Results

### 3.1. Orthologues of major genes involved in mammalian insulin secretion exist in zebrafish

Key proteins involved in the electrical coupling of glucose metabolism to insulin secretion in mammals include glucose transporters (GLUT2), K_ATP_ channels and VDCCs (electronic supplementary material, figure S1*a*). Orthologues for each of these are present in the zebrafish genome, and predicted K_ATP_ channel subunit sequences are highly conserved (ClustalW alignments, electronic supplementary material, figure S1*b,c*). In mammals, K_ATP_ channels are generated as octameric complexes of four pore-forming Kir6.x subunits and four accessory sulfonylurea receptor (SURx) subunits [22]; Kir6.2 and SUR1 form K_ATP_ channels in pancreatic islets and in the central nervous system, whereas Kir6.1 and SUR2 form K_ATP_ channels in smooth and striated muscles [22]. The genes for Kir6.2 (*KCNJ11*) and SUR1 (*ABCC8*) are immediately adjacent to one another in both zebrafish and human chromosomes (chromosome 25 in zebrafish and 11 in humans). The genes for Kir6.1 (*KCNJ8*) and SUR2 (*ABCC9*) are also adjacent to one another in both species (located on chromosome 4 in zebrafish and 12 in humans). Kir6.1, Kir6.2, SUR1 and SUR2 all show more than 70% amino acid identity between humans and zebrafish [9] (electronic supplementary material, figure S1*b*), with functional domains in SUR1 being highly conserved (electronic supplementary material, figure S3). Kir6.3, a pore-forming subunit unique to zebrafish and likely to be derived from zebrafish Kir6.2 in a duplication event, is located on zebrafish chromosome 15 and has no SUR gene in its vicinity. Kir6.3 and SUR1 expression have previously been described in the zebrafish central nervous system [9], but expression of the different K_ATP_ channel subunits has not been examined in other tissues.

### 3.2. K_ATP_ channels regulate glucose homeostasis in adult zebrafish

We initially probed the glucose metabolism, and the role of K_ATP_ in glucose control, in adult zebrafish using a glucose tolerance test. IP injection of glucose elevates blood glucose in adult zebrafish beyond vehicle alone, and glucose gradually normalizes (figure 1*a*, blue dotted line). As shown in figure 1*a*, co-injection of diazoxide along with glucose significantly slows the return of glucose to baseline. These results are consistent with the effect of diazoxide on IP glucose tolerance in mammals, and with previous experiments on larval zebrafish [7].

### 3.3. Zebrafish β-cells express functional K_ATP_ channels

To examine K_ATP_ channel expression and function in zebrafish β-cells, we have developed approaches to isolate islets and individual β-cells for gene expression as well as whole-cell and excised-patch voltage-clamp techniques (figure 1*b*). Fish that express eGFP using the zebrafish insulin promoter allowed isolation and dispersion of pancreatic islets to yield individual β-cells as described in methods [15] (figure 1*b*). Whole-cell patch clamp of these isolated zebrafish β-cells (figure 1*c*), exhibited rapidly declining voltage-activated currents above ∼−40 mV, and activation of large, almost linear potassium conductances that are maximal within a few minutes after initial dialysis of the cell with zero ATP solution. The weak inward rectification (evident above approx. 0 mV, figure 1*c*, left), and reversal potential very close to E_K_ (−80 mV, figure 1*c*, right), as well as the amplitude of this conductance, are indistinguishable from typical K_ATP_ currents activated in mammalian β-cells [23].

**Figure 2.**
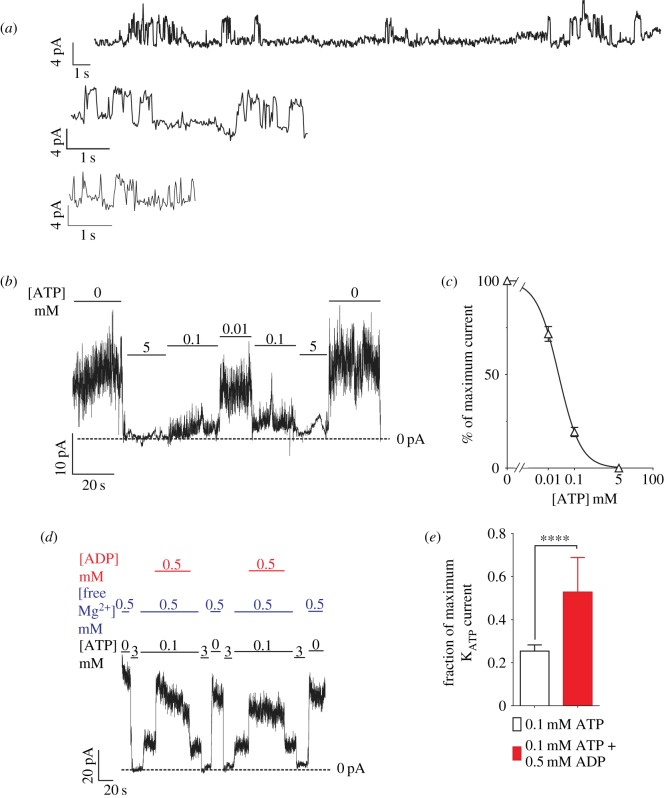
Excised-patch clamp reveals functional properties of K_ATP_ channels in zebrafish β-cells. (*a*) Individual K_ATP_ channels (4.35 pA at −50 mV) are detected in three representative membrane patches excised from zebrafish β-cells. (*b*) These K^+^ currents are inhibited by ATP, with (*c*) IC_50_ = 22.6 mM, *n*_H_ = 1.01. (*d*) These channels are also activated by increasing [Mg-ADP], quantified in (*e*). ^****^p < 0.0001 (Mann–Whitney test). The dose–response curve was generated from 16 cells derived from six pools of zebrafish islet (biological replicates). The ADP response graph comprises nine cells derived from four biological replicates. (*a,b* and *d*) Representative traces.

Inside-out excised-patch voltage-clamp experiments on zebrafish β-cells reveal potassium channels with single channel conductance of approximately 87 pS (4.35 pA at −50 mV driving force; figure 2*a*). Again, this property is indistinguishable that of from mammalian K_ATP_ channels formed form Kir6.2 + SUR1 subunits [24,25]. These channels are inhibited by increasing concentrations of ATP at the intracellular surface (figure 2*b,c*), with IC_50_ of 22.6 µM (*n*_H_ _=_ 1.01), very similar to reported values for mammalian β-cell K_ATP_ channels in the same conditions (IC_50_ ∼ 10–20 µM [21,24,26,27]). Furthermore, these channels are activated by addition of Mg-ADP to the cytoplasmic face (figure 2*d,e*), again similar to properties of mammalian K_ATP_ channels [28]. Taken together, these data show that zebrafish β-cells express functional K_ATP_ channels with activation and inhibition properties that are essentially the same as those expressed in mammalian β-cell K_ATP_ channels.

### 3.4. Zebrafish β-cell K_ATP_ channels show similar subunit composition and pharmacology to mammalian β-cell K_ATP_ channels

**Figure 3.**
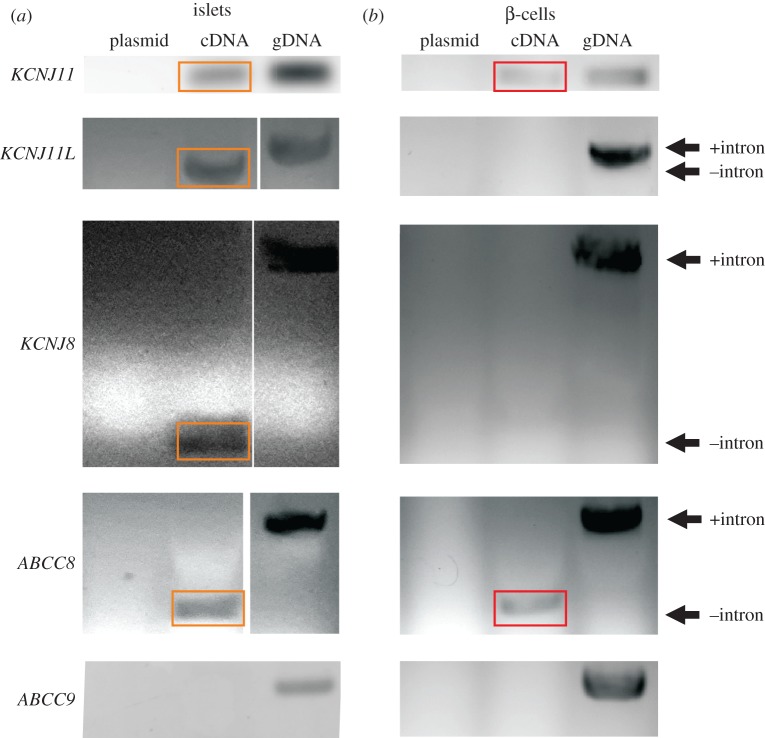
Zebrafish β-cell K_ATP_ channels are similar in composition to mammalian β-cell K_ATP_ channels. (*a*) PCR of RNA-derived cDNA from different pools of islets shows bands for orthologues of *KCNJ11* (Kir6.2)*, KCNJ8* (Kir6.1)*, KCNJ11 L* (Kir6.3) and *ABCC8* (SUR1), but no bands for *ABCC9* (SUR2). Plasmid DNA is a negative control for non-specific replication by primer mix; genomic DNA (gDNA) is a positive control for presence of target genes. These reactions were repeated over *n *≥ 5 separate pools of cDNA and gDNA for validation. Primers for *KCNJ11 L*, *KCNJ8* and *ABCC8* span exons to distinguish gDNA from cDNA. There are no introns in *KCNJ11*. (*b*) PCR of RNA-derived cDNA from eGFP-sorted β-cells shows bands for orthologues of *KCNJ11* and *ABCC8*, but no bands for the other subunits. Images were cropped and resized, and in some cases contrast-enhanced to improve clarity. Original images are in the electronic supplementary material, figures S4 and S5. Orange and red boxes highlight transcript presence in islet and β-cell cDNAs, respectively.

We performed PCR on genomic DNA (gDNA) and cDNA generated from RNA isolated from zebrafish islets, to characterize Kir6 and SUR subunit expression in zebrafish. Genes for Kir6.1, Kir6.2, Kir6.3, SUR1 and SUR2 were all detected in gDNA but only Kir6.2, Kir6.1, Kir6.3 and SUR1 were consistently detected in islet cDNA (figure 3*a*). The similarity of zebrafish K_ATP_ currents to those expressed in mammalian β-cells is consistent with both being formed of Kir6.2 and SUR1 subunits, raising the question of the relevance of Kir6.1 and Kir6.3 expression, Kir6.3 having been detected in fish neurons by RNA *in situ* hybridization studies [9]. cDNA derived from eGFP-sorted β-cells indicates transcription of only *KCNJ11* (Kir6.2) and *ABCC8* (SUR1) (figure 3). While β-cells form the majority of cells in the islet, islets are innervated and permeated by capillaries [29]; the presence of *KCNJ8* (Kir6.1) and *KCNJ11 L* (Kir6.3) transcripts in whole islets may reflect the presence of these other cell types.

**Figure 4.**
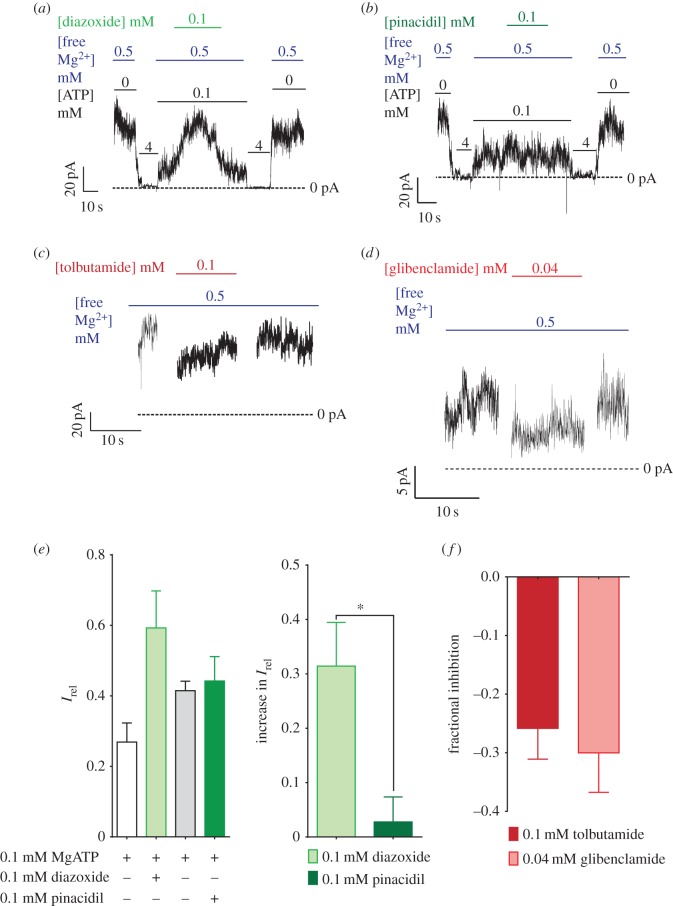
Zebrafish β-cell K_ATP_ channels exhibit similar in pharmacology to mammalian β-cell K_ATP_ channels. Zebrafish β-cell K_ATP_ channels show activation by diazoxide (*a*) but not by pinacidil (*b*) in excised patches. These channels also show inhibition by both tolbutamide (*c*) and glibenclamide (*d*). (*e,f*) Quantification of activation (*e*) and inhibition (*f*) of the K_ATP_ channels. Asterisk indicates *p* < 0.05 by the Mann–Whitney test. In (*e*), the right panel indicates the increase in *I*_rel_ produced by each drug, whereas the left panel indicates the fraction of overall maximum current in each condition. These are quantified from recordings of six cells for diazoxide, four cells for pinacidil and glibenclamide, and five cells for tolbutamide.

Mammalian SUR subunits respond differentially to activator and inhibitor compounds: the potassium channel opener (KCO) diazoxide is a more effective activator of SUR1-containing K_ATP_ channels and pinacidil is a more effective activator of SUR2-containing channels [30,31]. Sulfonylureas, furthermore, typically close SUR1-containing K_ATP_ channels approximately 100- to 1000-fold more effectively than SUR2-containing K_ATP_ channels in mammals [32]. Residues involved in drug sensitivity are conserved between zebrafish and mammalian SUR subunits (electronic supplementary material, figures S2 and S3). In excised zebrafish β-cell membranes, addition of Mg^2+^ and diazoxide is sufficient to activate K_ATP_ channels (figure 4*a,e*), whereas pinacidil is ineffective (figure 4*b*) at the same concentration. Two sulfonylurea drugs, tolbutamide (figure 4*f*) and glibenclamide (figure 4*f*), both inhibit the zebrafish β-cell K_ATP_ at relatively low concentrations in excised patches, similar to the level of inhibition seen for mammalian Kir6.2+SUR1 channels at the same drug concentrations in similar conditions [33]. Potent response to sulfonylureas and diazoxide, but not to pinacidil, is consistent with the expression data showing that SUR1 is the only SUR detected in islet cDNA. Taken together, these data indicate that the subunits comprising zebrafish β-cell K_ATP_ channels and the drug responsivity of these channels are essentially the same as their mammalian counterparts.

## 4. Discussion

### 4.1. Structure and functional properties of β-cell K_ATP_ channels are conserved between zebrafish and mammals

Mammalian K_ATP_ represents a family of potassium channels generated by various combinations of Kir6.1/2 and SUR1/2 subunits [34,35]. Expression patterns and functional properties have been extensively characterized, and shown to be generally well conserved between mammalian species [4,21,22,36–38]. K_ATP_ channel subunit orthologues are clearly present in all sequenced vertebrate genomes, but there have been surprisingly few studies of structural or functional properties of K_ATP_ channels in islets from non-mammalian vertebrate classes. Studies of K_ATP_ structure and function in fishes have been very limited, in part, owing to technical difficulties of identification and isolation of specific cell types. Zhang *et al*. identified a third, unique, pore-forming subunit, Kir6.3 [9], in zebrafish, and showed that this subunit is expressed in the central nervous system via RNA-*in situ* hybridization, but expression and potential roles in other tissues were not explored. Here, using fluorescently tagged β-cells in transparent Casper fish, we have succeeded in efficiently identifying, isolating and dissociating zebrafish islets. We show that zebrafish β-cells express functional K_ATP_ channels that exhibit very similar composition (Kir6.2 and SUR1) and pharmacology (activation by diazoxide, but not pinacidil) to those in mammalian β-cells, and that modulation of these channels affects adult fish glucose homeostasis similarly to the effects in mammals.

### 4.2. Conservation of K_ATP_ channel-dependent insulin secretion mechanisms between teleost fishes and mammals

Rapid responses to metabolic changes are challenges faced by all organisms, and the potential importance of insulin signalling in such responses is highlighted by the high conservation of insulin structure and insulin signalling pathways across vertebrates and invertebrates, with evolutionary lineages that diverged long ago [39,40]. However, the last common ancestor between teleost fishes and humans is estimated to have lived approximately 450 million years ago [41], and while insulin and other hormones are structurally conserved across the vertebrates, whether secretory regulation and functional consequences are as conserved is less clear.

The finely tuned properties and regulatory features of β-cell K_ATP_ channels are absolutely key to the regulation of mammalian insulin secretion [2,4]. A role for K_ATP_ channels in modulating glucose metabolism in zebrafish has been implied by the demonstration that treatment of fish larvae with the K_ATP_ opener diazoxide increases whole-larval glucose and that the inhibitor glibenclamide lowers whole-larval glucose [7]. Transgenic overexpression of mammalian K_ATP_ channels with gain-of-function mutations was also sufficient to increase larval glucose in these studies, whereas a dominant-negative mammalian K_ATP_ channel lowered larval glucose [7]. It has also been suggested that diazoxide interferes with regeneration of pancreatic islets in fishes after alloxan treatment, while glimepiride, a sulfonylurea, enhances recovery [10]. However, despite implicating K_ATP_ in metabolic control, none of these earlier studies characterized the properties of native zebrafish islet K_ATP_ channels or directly assessed their role in insulin secretion. We are unaware of *ex vivo* analyses of insulin secretion in zebrafish islets, but our findings suggest that K_ATP_ channels are conserved in both functional expression and properties between zebrafish and mammalian β-cells (figures 2, 3 and 4). Manipulation of K_ATP_ channels *in vivo* with the pharmacological activator diazoxide suggests that K_ATP_ channels are also key to normal glucose tolerance in adult fishes (figure 1*a*).

### 4.3. Zebrafish as model organisms for studying metabolic diseases

In addition to their use in tracking temporal expression patterns of transcription factors in endocrine development, zebrafish have been used to model atherosclerosis, the consequences of high fat diet feeding, hyperglycaemia and other metabolic interventions, as well as the regeneration of key endocrine organs, including the pancreas [42]. Our finding that zebrafish β-cells express functional K_ATP_ channels with very similar biophysical properties and pharmacology to mammalian channels, and that channel activation significantly impairs whole-body glucose clearance in adult fish, will lend further support to the use of these animals to model metabolic diseases.

Forward genetic screens can be powerful tools for unmasking subtle modifiers of disease phenotypes, but infrequent reproduction, low litter numbers, long maturation time and high cost limit the use of mammalian species for such studies. Zebrafish reproduce frequently and with large clutches (potentially hundreds of embryos per clutch), allowing analysis of thousands of individuals in short periods. The genome is fully sequenced, and techniques for introducing mutations in zebrafish have been well streamlined [43,44]. Larvae are transparent, allowing easy visualization of genetic markers or fluorescent dyes [14,45]. Zebrafish develop metabolic abnormalities when fed high-fat diets [46,47], show similar complications of persistently high glucose and have many of the same transcription factor pathways involved in development of endocrine, liver and other organs important in controlling metabolism [44,48]. While zebrafish may thus offer major advantages for screening diabetes modifiers, details of comparative organ biology must first be evaluated and further studies like those we describe here are required.

## Supplementary Material

Supplementary Figures

## Supplementary Material

Supplementary data and original records

## Data accessibility

Our data are deposited at Dryad Digital Repository: http://dx.doi.org/10.5061/dryad.1v074 [49]. Electronic supplementary material, including figures, legends and additional results, are also published on the Royal Society Open Science journal site.
