# Supplementary material for: Expression and function of ATP-dependent potassium channels in zebrafish islet β-cells
Source: R Soc Open Sci. 2017 Feb 8;4(2):160808. doi: 10.1098/rsos.160808 (PMC5367309; doi:10.1098/rsos.160808)
Supplement: Supplementary Figures [file rsos160808supp1.pdf]

A

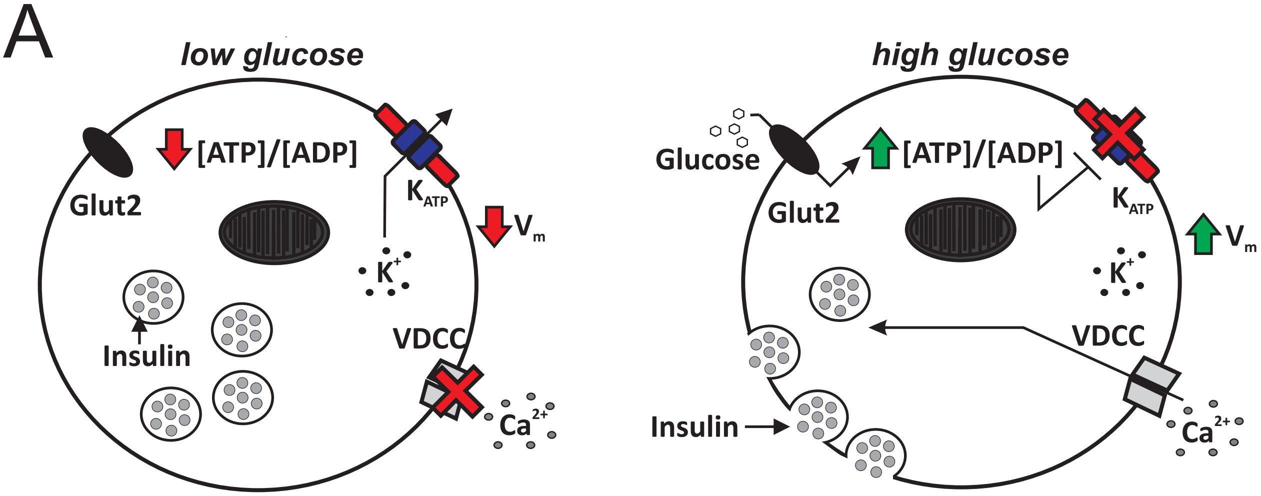

B

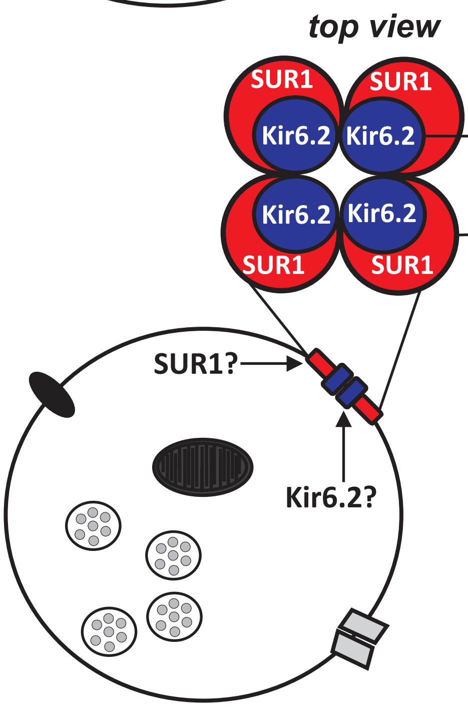

	zKir6.1	zKir6.2	zKir6.3	hKir6.1	hKir6.2
zKir6.1	100.0	67.2	58.1	80.0	67.1
zKir6.2		100.0	65.1	68.0	72.5
zKir6.3			100.0	61.3	64.5
hKir6.1				100.0	69.7
hKir6.2					100.0

	zSUR1	zSUR2	hSUR1	hSUR2A	hSUR2B
zSUR1	100.0	65.9	75.5	66.0	67.3
zSUR2		100.0	65.9	79.0	78.4
hSUR1			100.0	66.8	67.8
hSUR2A				100.0	98.3
hSUR2B					100.0

% Amino Acid Identity

Low — High

C

	zKir6.1	zKir6.2	zKir6.3	hKir6.1	hKir6.2
zKir6.1	100.0	67.7	60.3	71.4	71.6
zKir6.2		100.0	64.1	67.4	69.8
zKir6.3			100.0	60.6	63.7
hKir6.1				100.0	69.5
hKir6.2					100.0

	zSUR1	zSUR2	hSUR1	hSUR2A	hSUR2B
zSUR1	100.0	66.4	71.7	65.8	66.3
zSUR2		100.0	66.4	71.7	71.2
hSUR1			100.0	65.1	64.5
hSUR2A				100.0	98.6
hSUR2B					100.0

% Nucleotide Identity

Low — High

Supplemental Figure 1

```
Majority     MLXRKGIIPEEYXLTRLAED-PRKPRXRDRQRKARFIAKSGACNVAHKNIREQGRFLQDVFTTLVDLKWRHTLVIFTMSF
                      10        20        30        40        50        60        70
zKir6.2prot  MLSRKGLIPEDYLLTRLAED-VLQPKFKTKQRKARFVAKNGTCNVAHTNIREQGRFLQDVFTTLVDLKWLHTLVIFTMSF  79
hKir6.2prot  MLSRKGIIPEEYVLTRLAED-PAEPRYRARQRRARFVSKKGNCNVAHKNIREQGRFLQDVFTTLVDLKWPHTLLIFTMSF  79
zKir6.1prot  MLPRKSIIPEEFALPALVSRVPRKPVFRDRVNKARFIAKSGACNLAHKNIREQGRFLQDVFTTLVDLKWRFTLVIFTMTF  80
zKir6.3prot  MLARKGLLPDGFLLTRLAED-AQPSRIRPKSQRARFITKSGSCNVAHKNIREQGRFLQDVFTTMVDLKWQHSLLIFTSAF  79
hKir6.1prot  MLARKSIIPEEYVLARIAAENLRKPRIRDRLPKARFIAKSGACNLAHKNIREQGRFLQDIFTTLVDLKWRHTLVIFTMSF  80

Majority     LCSWLLFAMXWWLXAFAHGDLDPXX--XPGXEP-------CVTXIHSFTSAFLFSIEVQVTIGFGGRMXTEECPLAIXXL
             80        90        100                10        120       130       140
zKir6.2prot  LCSWLLFGMIWWLIAFAHGDLDLKG---EDFVP-------CVTDIHSFSSAFLFSIEVQVTIGFGGRMITEECVSAIVIL  149
hKir6.2prot  LCSWLLFAMAWWLIAFAHGDLAPSE---GTAEP-------CVTSIHSFSSAFLFSIEVQVTIGFGGRMVTEECPLAILIL  149
zKir6.1prot  LCSWLLFAMSWWLVAFGHGDLDTDR--KPAVEQ-------CVTNVNSFTSAFLFSIEVQVTIGFGGRMITERCPIAIAFL  151
zKir6.3prot  LCSWMLFAMVWWLLAFAHGDLEPRDPNEPGPVP-------CVTSIHSFTSAFLFSIEVQVTIGFGGRMVTEECPLAIIVL  152
hKir6.1prot  LCSWLLFAIMWWLVAFAHGDIYAYME-KSGMEKSGLESTVCVTNVRSFTSAFLFSIEVQVTIGFGGRMMTEECPLAITVL  159

Majority     ILQNIVGLIINAIMLGCIFMKTAQAHRRAETLIFSRHAVIAVRNGRLCFMFRVGDLRKSMIISATVXXQVVRKTXTPEGE
             150       160       170       180       190       200       210       220
zKir6.2prot  ILQNIVGLVINAIMLGCIFMKTAQANRRAETLIFSKHAVITVRNNKLCFMFRLGDLRKSMIISATVHMQVVRKTVTSEGE  229
hKir6.2prot  IVQNIVGLMINAIMLGCIFMKTAQAHRRAETLIFSKHAVIALRHGRLCFMLRVGDLRKSMIISATIHMQVVRKTTSPEGE  229
zKir6.1prot  ILQNIIGLIINAVMLGCIFMKTAQSHRRAETLIFSRHAVIAVRNNRLCFMIRVGDLRKSMIINAVVRLQVVRKTMTPEGE  231
zKir6.3prot  IIQNILGLIINAIMLGCVFMKTAQANRRAETLIFSRNAVIAPRNGRPTLMFRVGDLRKSMIISANIQLQVIRRTVTAEGE  232
hKir6.1prot  ILQNIVGLIINAVMLGCIFMKTAQAHRRAETLIFSRHAVIAVRNGKLCFMFRVGDLRKSMIISASVRIQVVKKTTTPEGE  239

Majority     VVPXHQXDIQXENPVGXNSIFLVAPLIIXHVIDKNSPLYDLSAXDLAH-XDLEVIVILEGVVETTGITTQARTSYXAEEI
             230       240       250       260       270       280       290       300
zKir6.2prot  MVPLDQIDIQMDNPVGTNSIFLVSPLIISHTIDKNSPLYDLSADDLLH-EDIEVIVVLEGVVETTGITTQARTSYLSEEI  308
hKir6.2prot  VVPLHQVDIPMENGVGGNSIFLVAPLIIYHVIDANSPLYDLAPSDLHHHQDLEIIVILEGVVETTGITTQARTSYLADEI  309
zKir6.1prot  VIPIQQIDVQTESAVAGNSIFLLAPLIICHVIDKDSPLYDLSAMELQC-SDLEVIVILEGVVETTGITTQARTSYVTEEI  310
zKir6.3prot  VIPVCQLDIQVENPLRSNGIFLVSPLIISHTIDRGSPLYELSAQSMAT-EDLEIIVILEGVVETTGITMQARTSYIPEEI  311
hKir6.1prot  VVPIHQLDIPVDNPIESNNIFLVAPLIICHVIDKRSPLYDISATDLAN-QDLEVIVILEGVVETTGITTQARTSYIAEEI  318

Majority     LWGHRFVPIVTEEDGXYSVDYSKFGNTVKVATPRCSARELDEKPSILIQTLQXSEX-------SXXXSLRKRXSXXXXXX
             310       320       330       340       350       360                370
zKir6.2prot  LWGHRFVPTVSEEDGMYAVDYSKFGNTVKVATPNSSARTLEEAGGMERFKLQETS--------GACAAVRRR--------  372
hKir6.2prot  LWGQRFVPIVAEEDGRYSVDYSKFGNTIKVPTPLCTARQLDEDHSLLEALTLAS----------ARGPLRKR-S------  372
zKir6.1prot  QWGHRFVPIVTEEEGVYSVDYSKFGNTVKVATPRCSARELDEKPSILIQTLQKSEL-------SHQNSLRKRNSMRRNNS  383
zKir6.3prot  LWGRRFVSIMTEEDGRYSVDYSKFGNTVPVRMPALSAKELDQTRGVQDSGPHEGKPQGWGLVRAGRGGYRRGGARACDDT  391
hKir6.1prot  QWGHRFVSIVTEEEGVYSVDYSKFGNTVKVAAPRCSARELDEKPSILIQTLQKSEL-------SHQNSLRKRNSMRRNNS  391

Majority     XXX------XX-XXXXSXLXVPKVXFMTPEGXXNX-X----
                                  380       390
zKir6.2prot  -----------------VKHPNTKEM                                                        381
hKir6.2prot  ----------------VPMAKAKPKFSISPDSLS                                                390
zKir6.1prot  MRK-----GNSMRRNNSALAVPKVQFLTPEGGANLAVT                                            416
zKir6.3prot  AAKPWYVPAEKEEEKKGQKKTVKLEEIGREIEEETTEDMSD                                         432
hKir6.1prot  MRR-----NNSIRRNNSSLMVPKVQFMTPEGNQNTSES                                            424
```

Supplemental figure 2

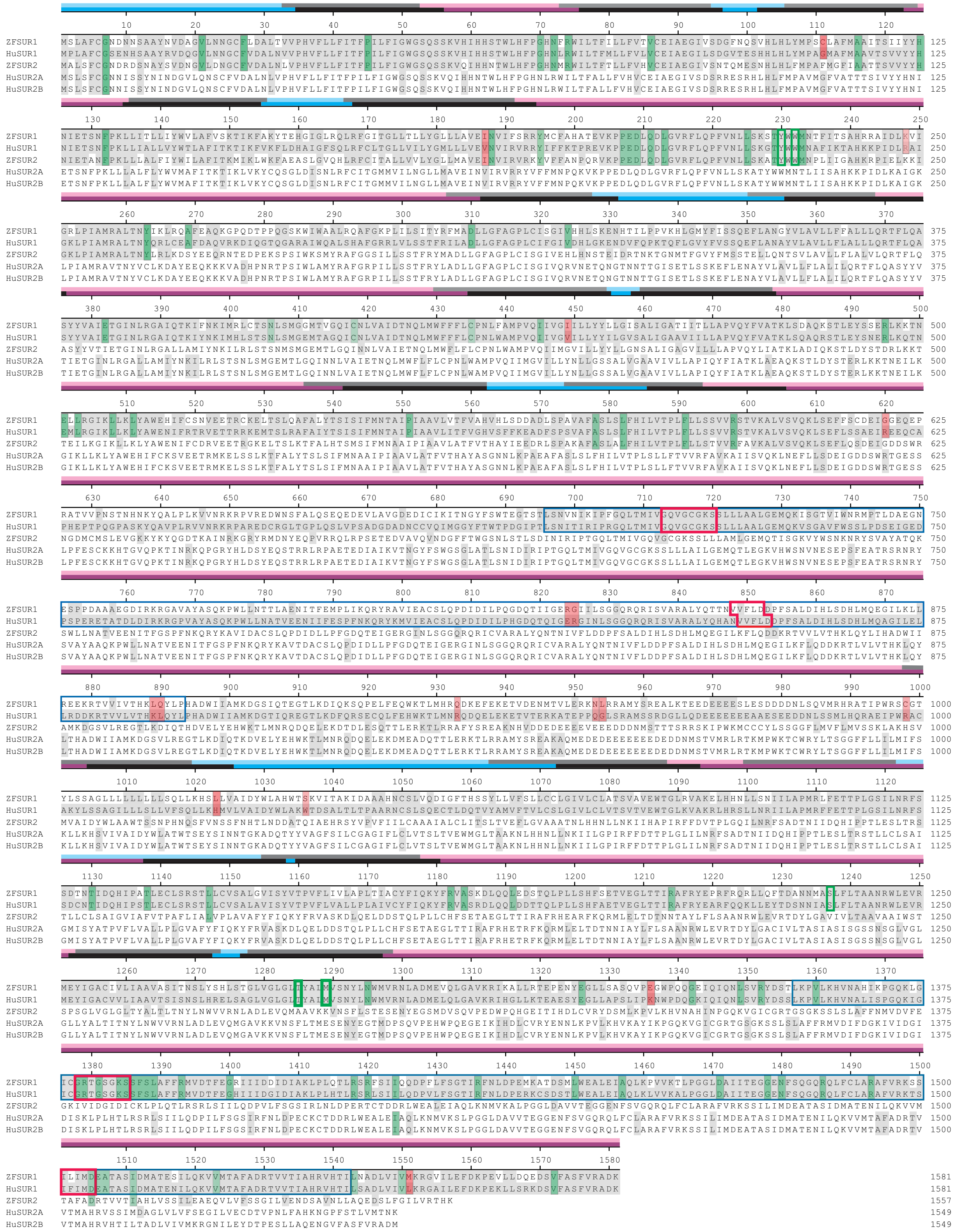

Supplemental figure 3

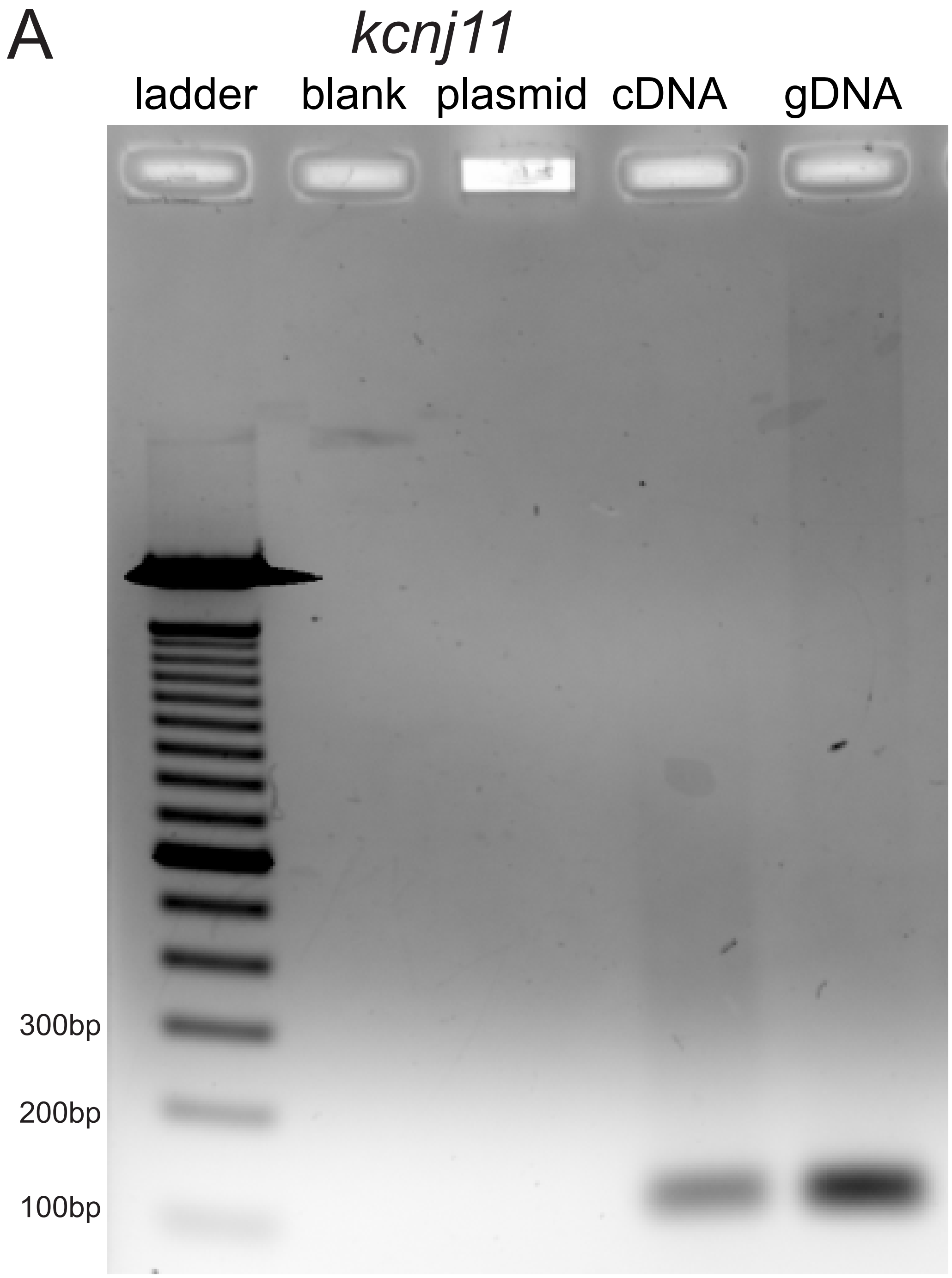

Supplemental figure 4

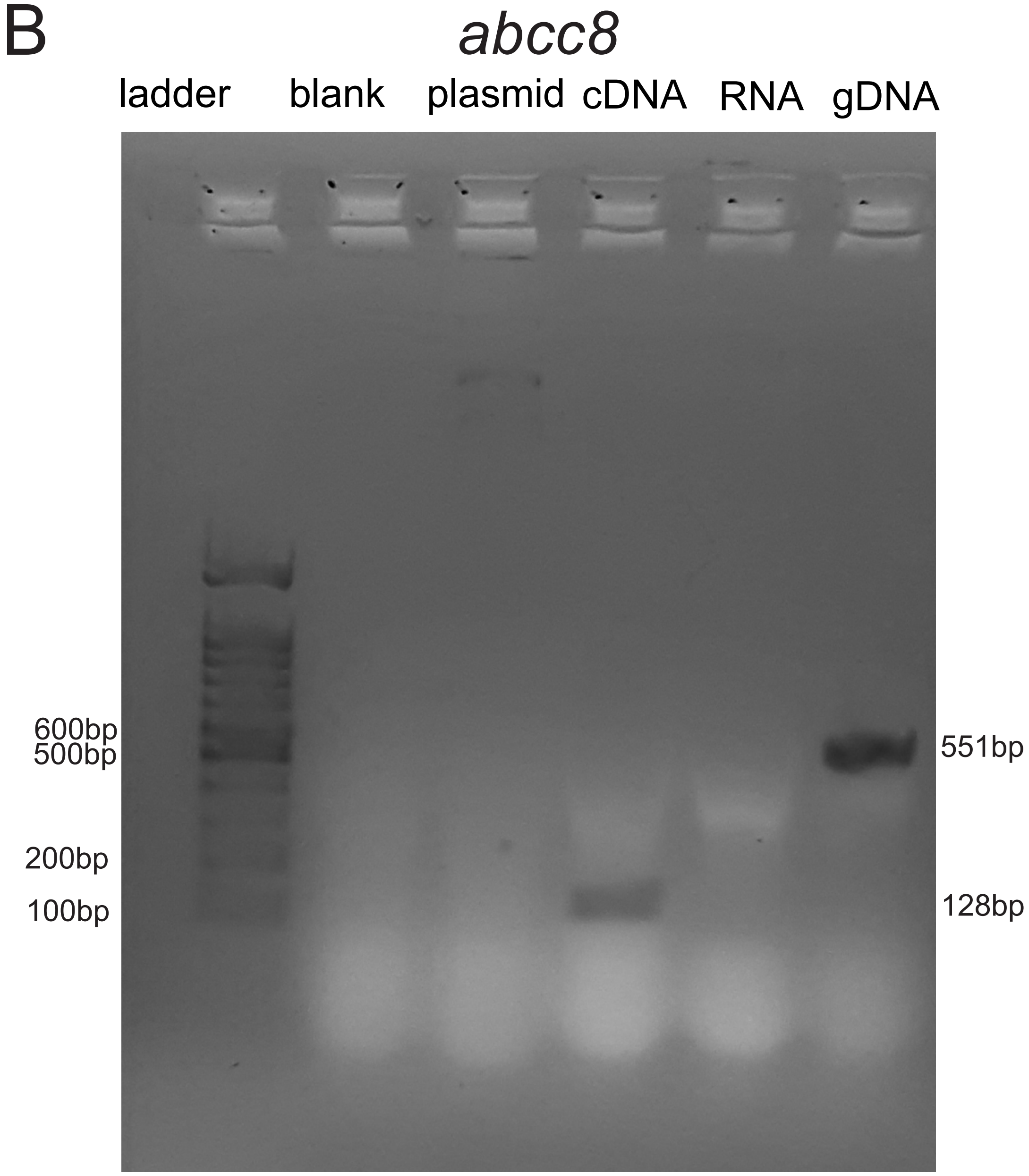

Supplemental figure 4

C

kcnj8

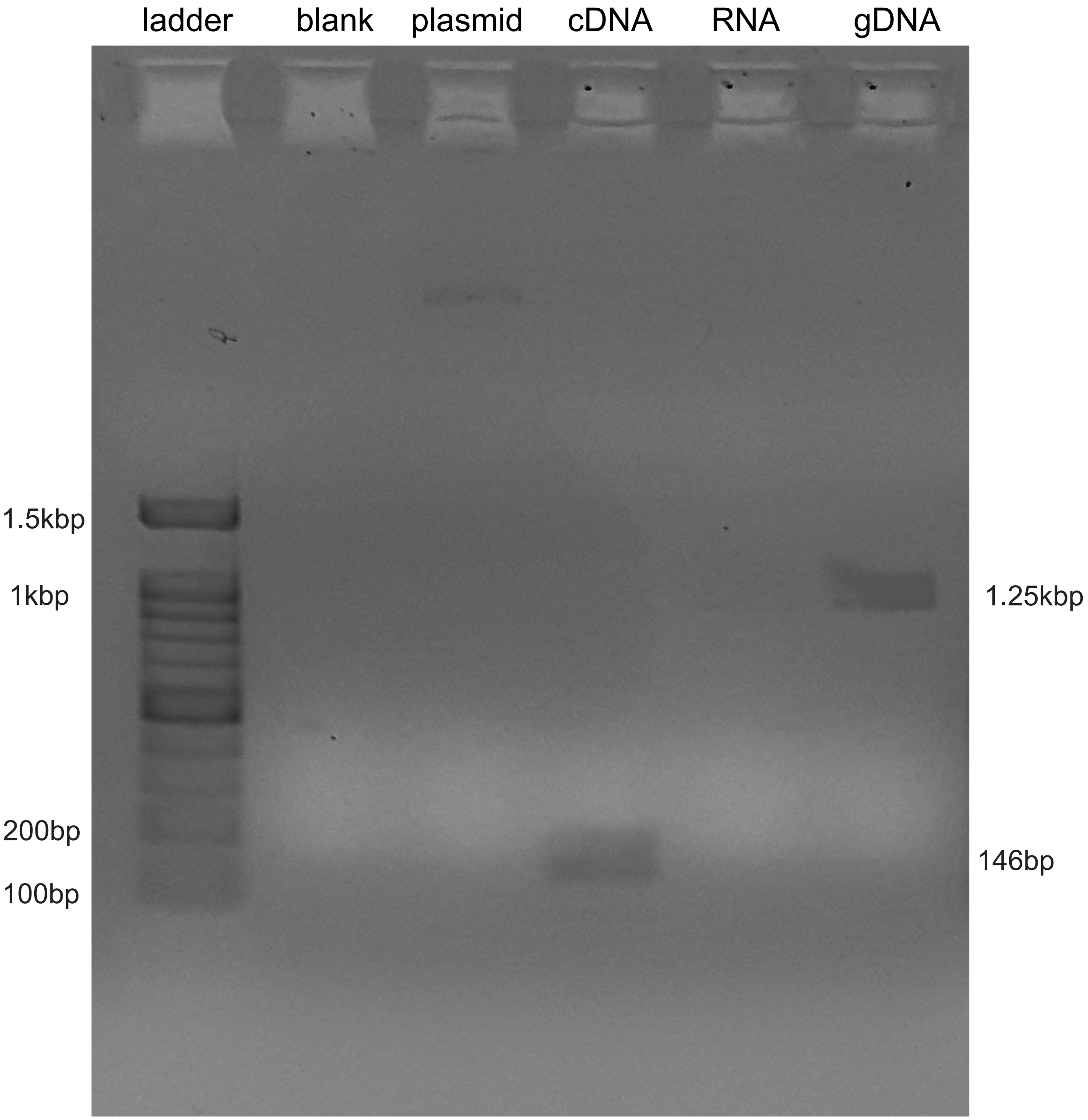

Supplemental figure 4

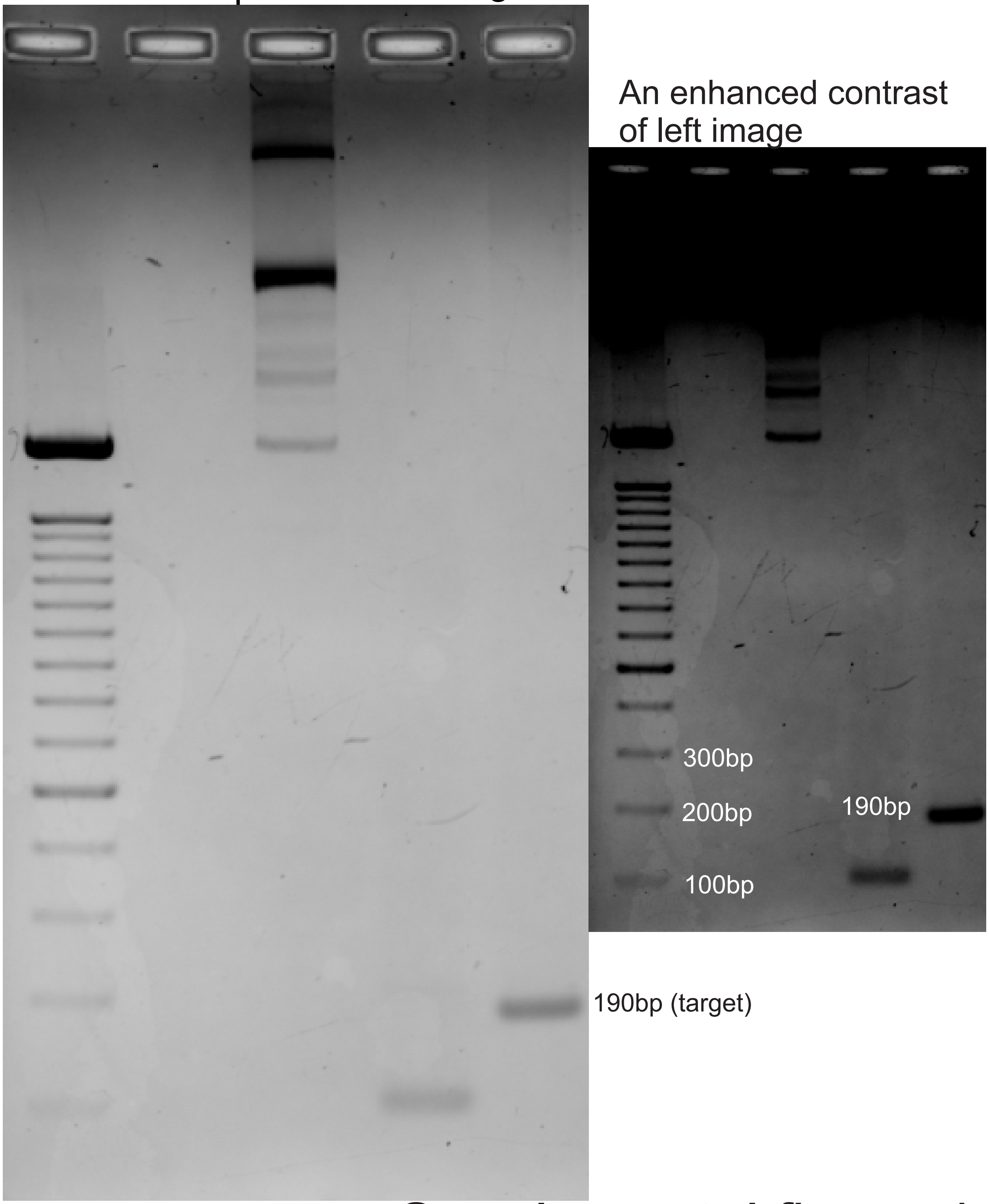

Supplemental figure 4

E

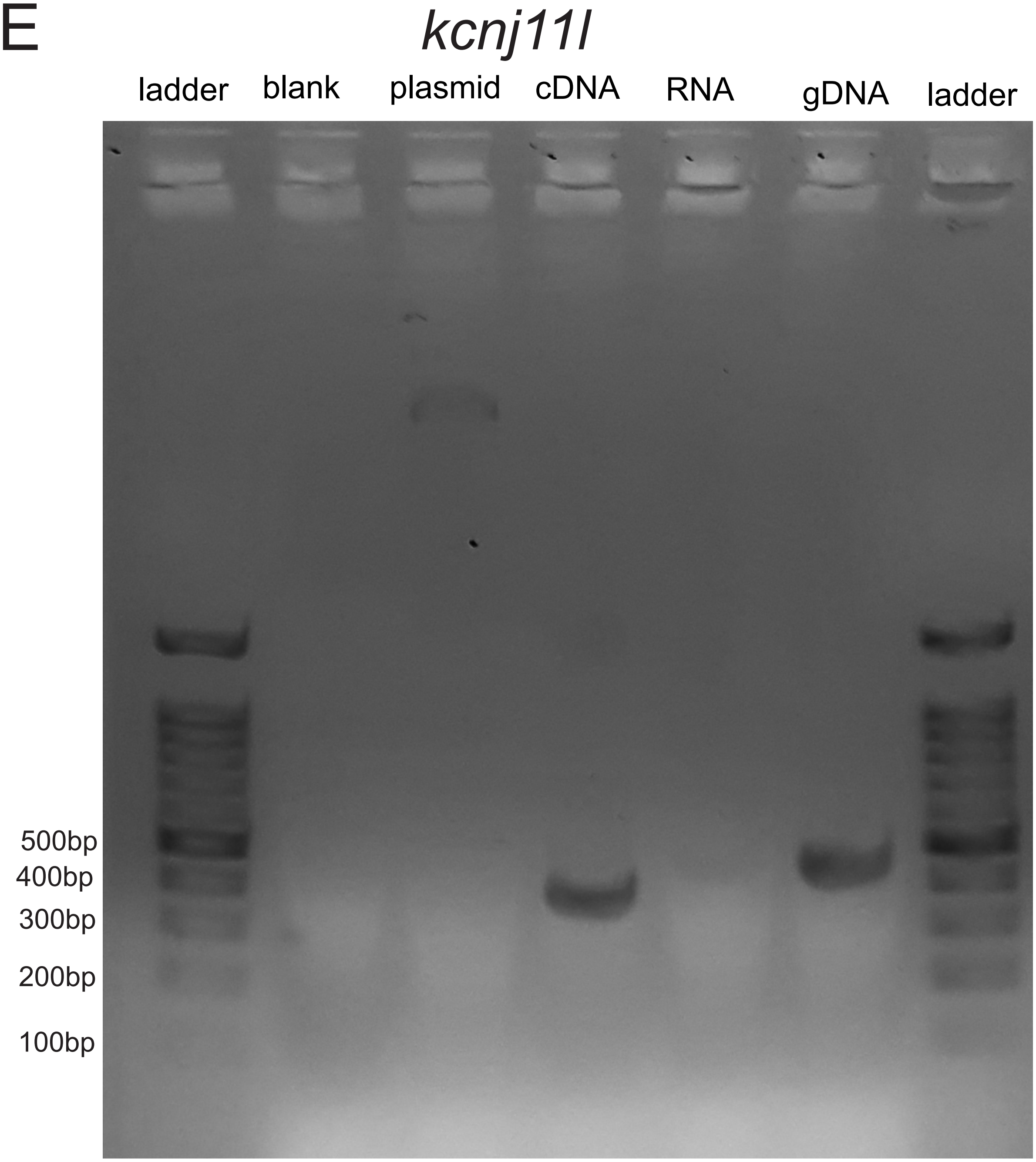

Supplemental figure 4

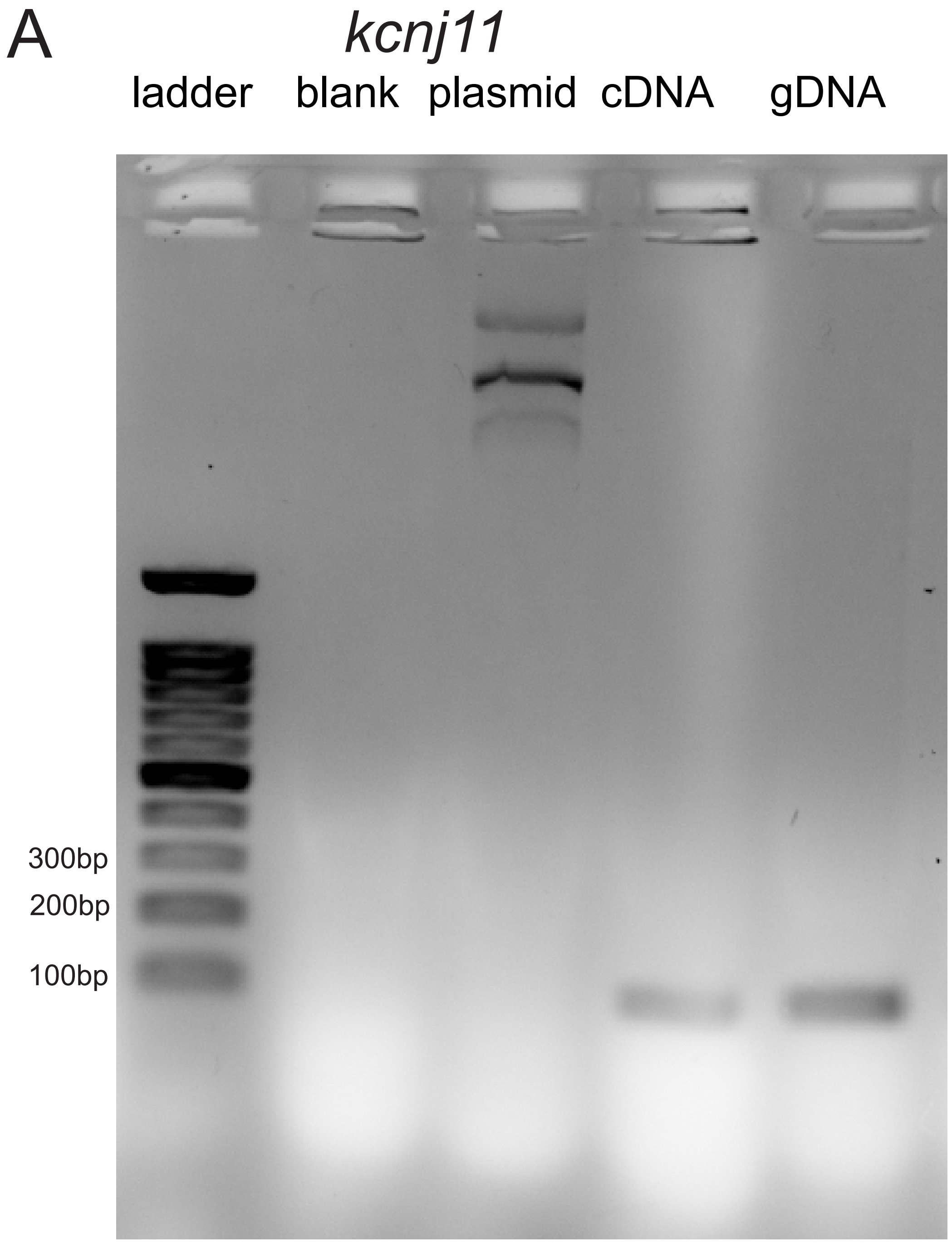

Supplemental figure 5

B

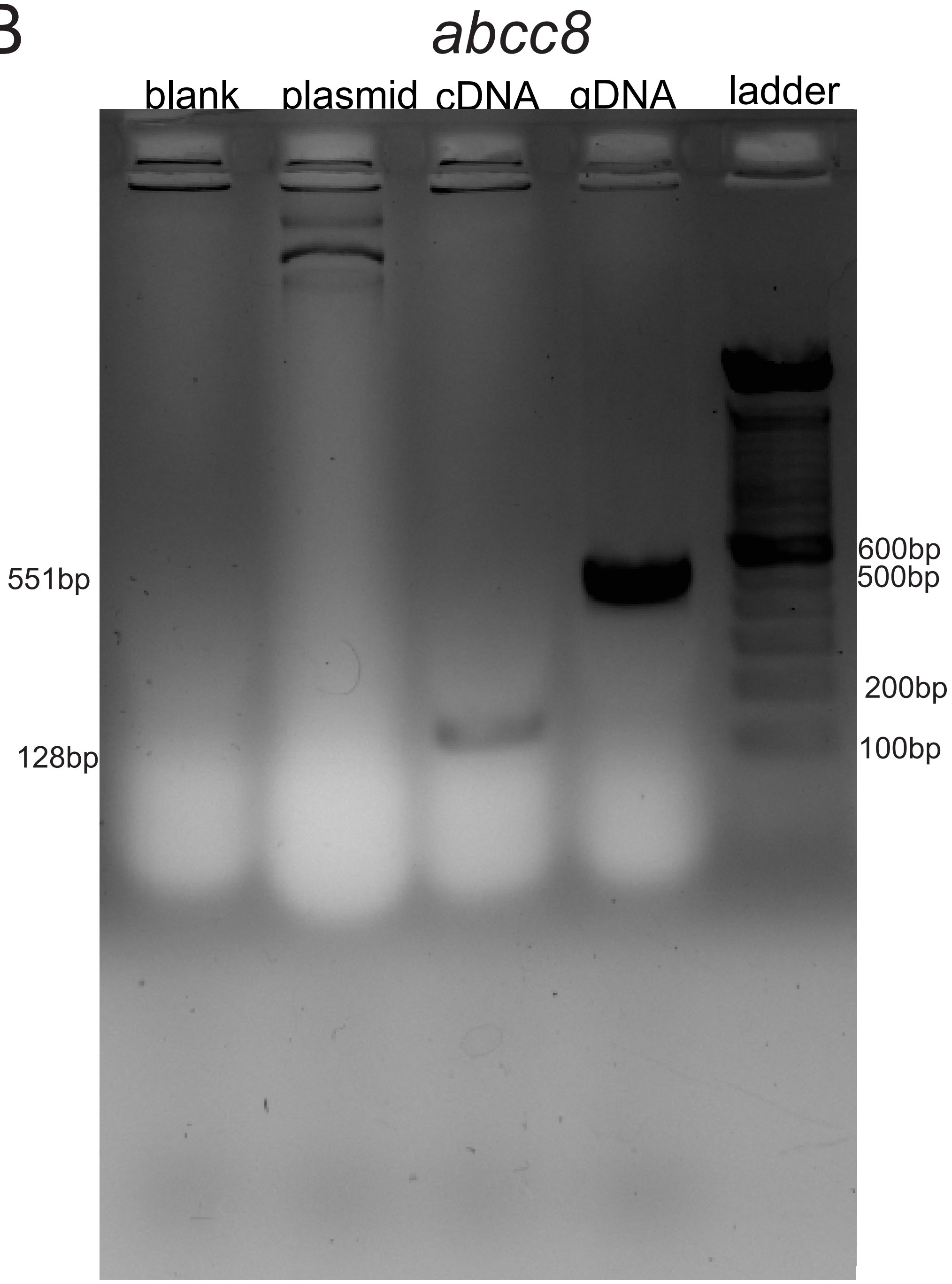

Supplemental figure 5

C

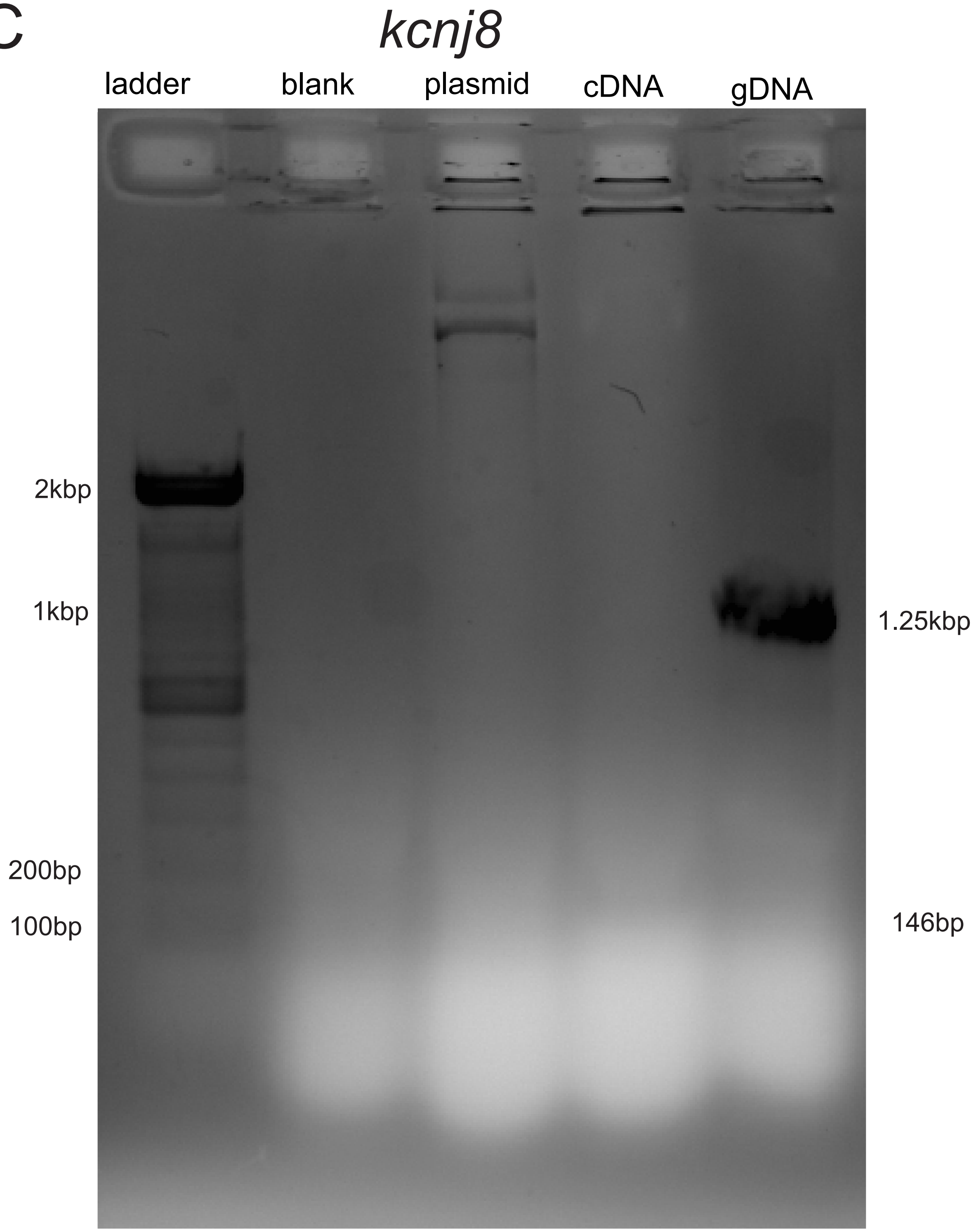

Supplemental figure 5

D

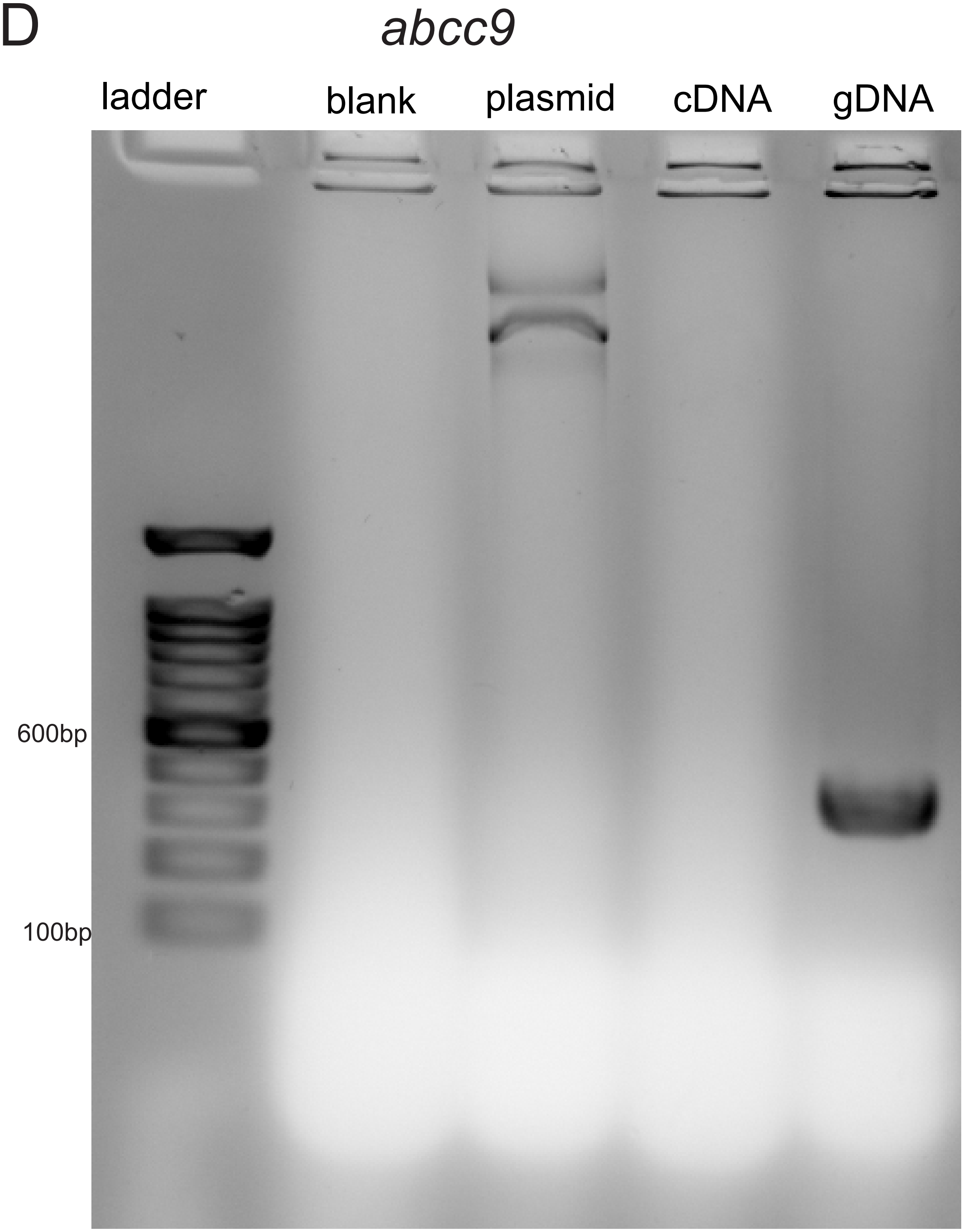

Supplemental figure 5

E

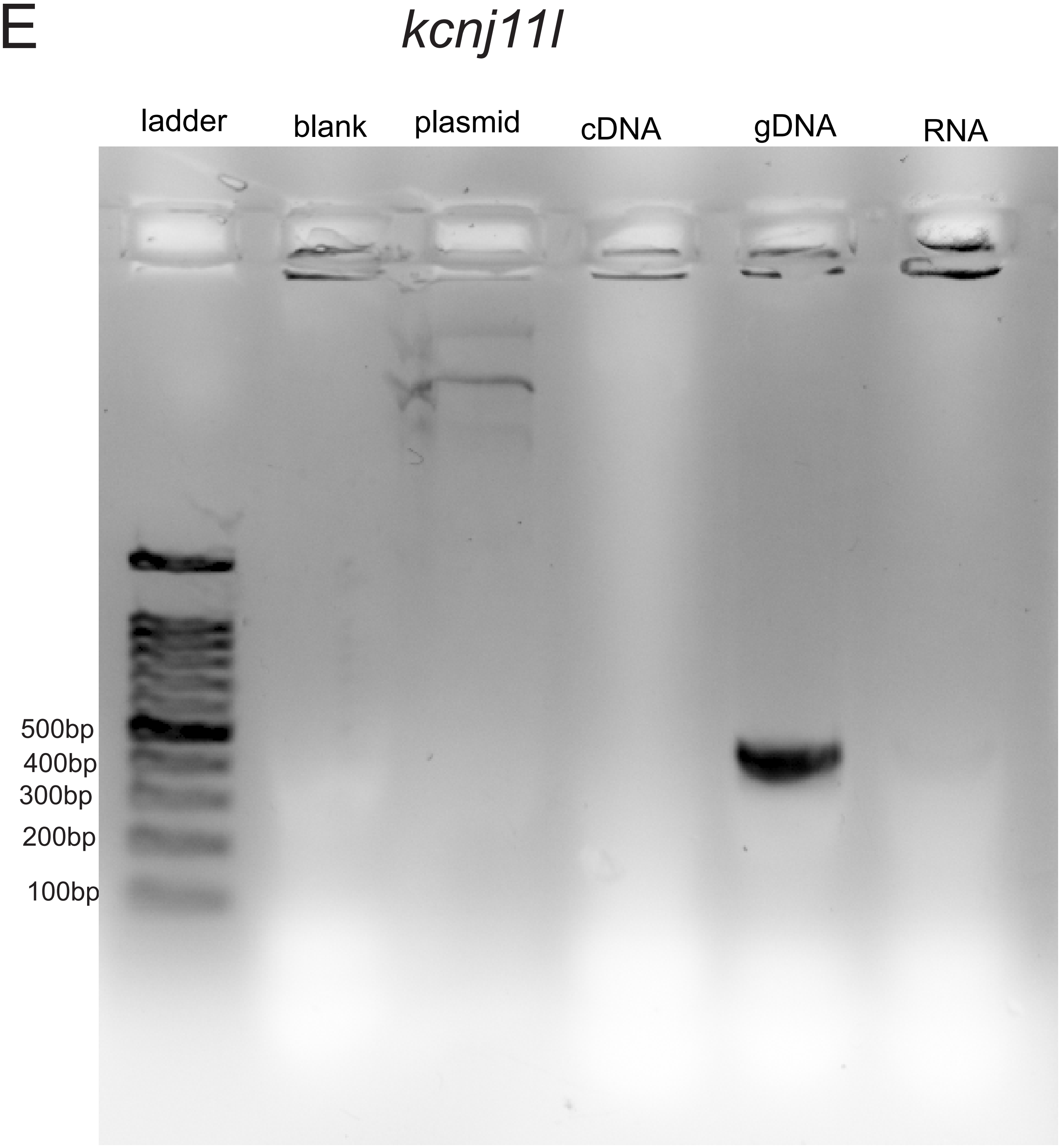

Supplemental figure 5

A

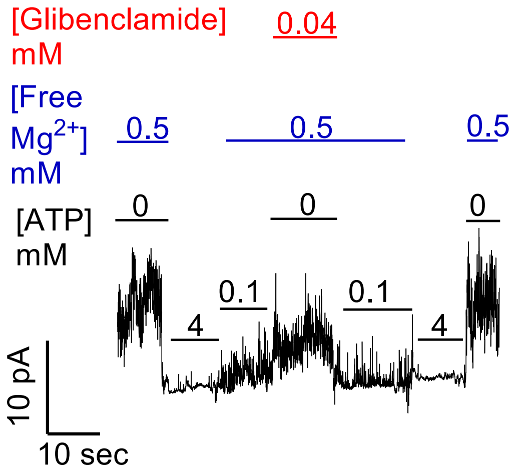

B

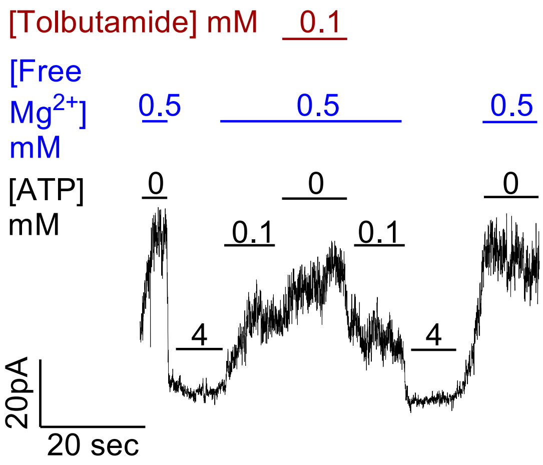

Supplemental Figure 6

A

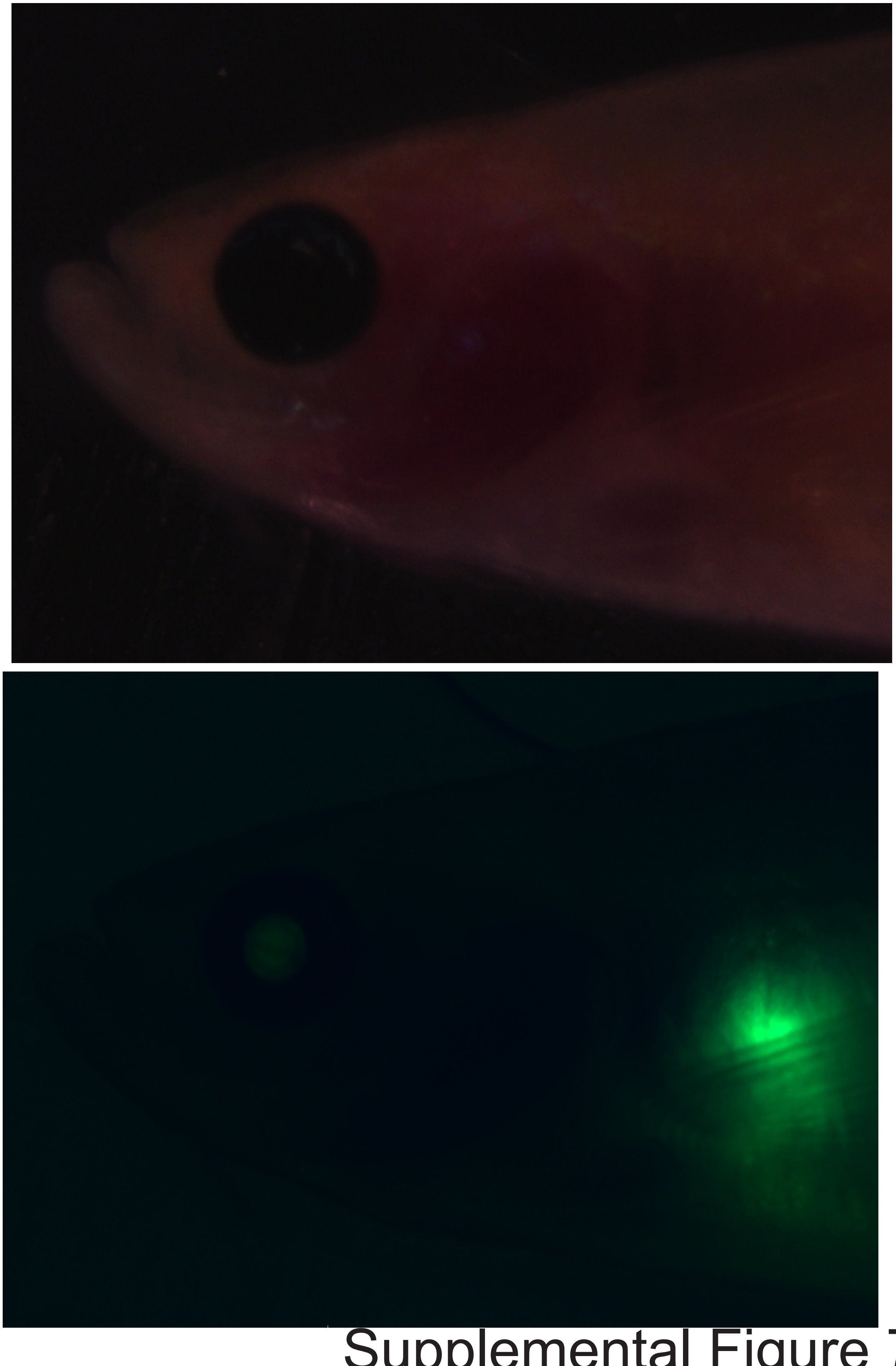

Supplemental Figure 7

B

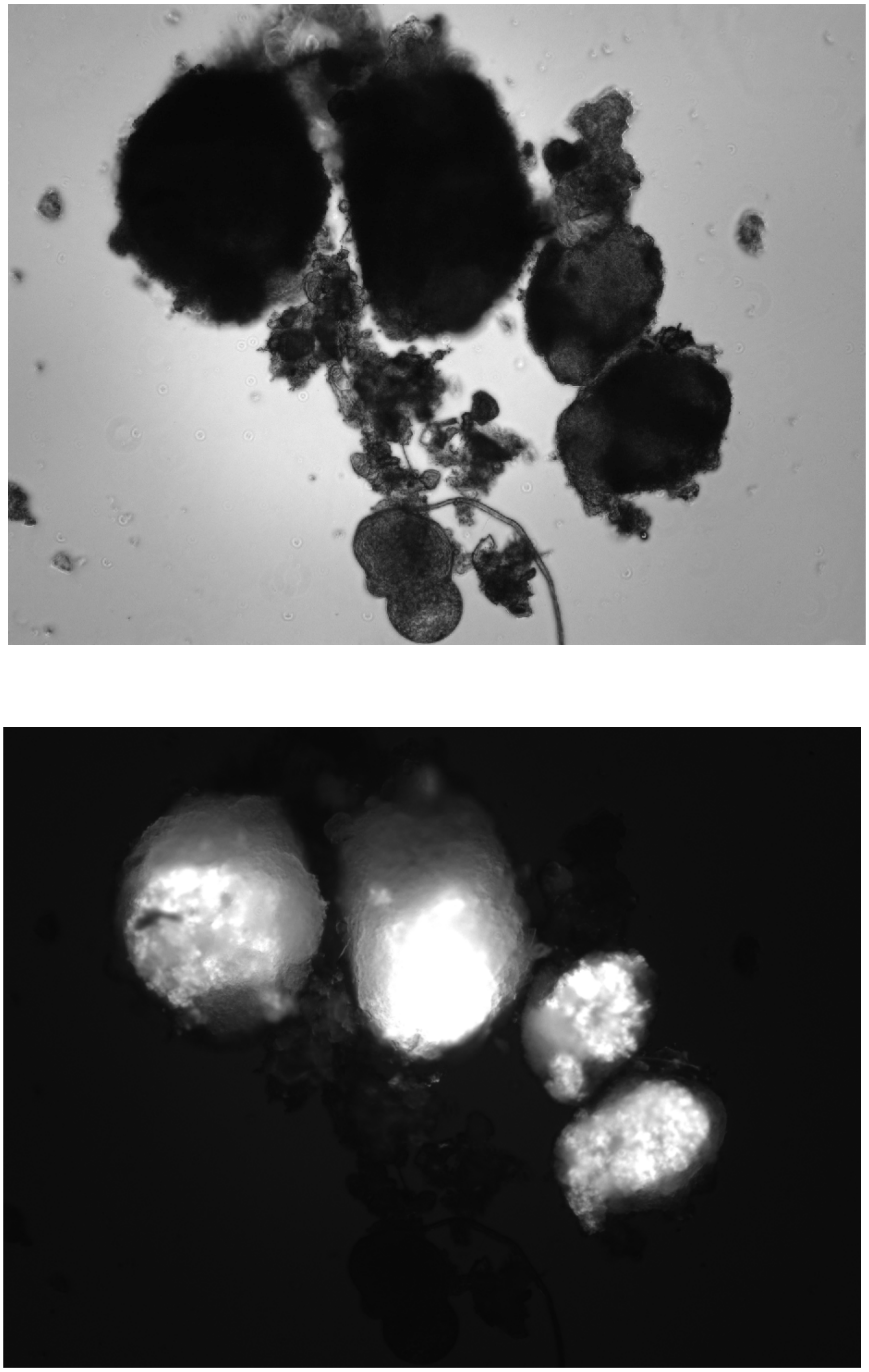

Supplemental Figure 7

C

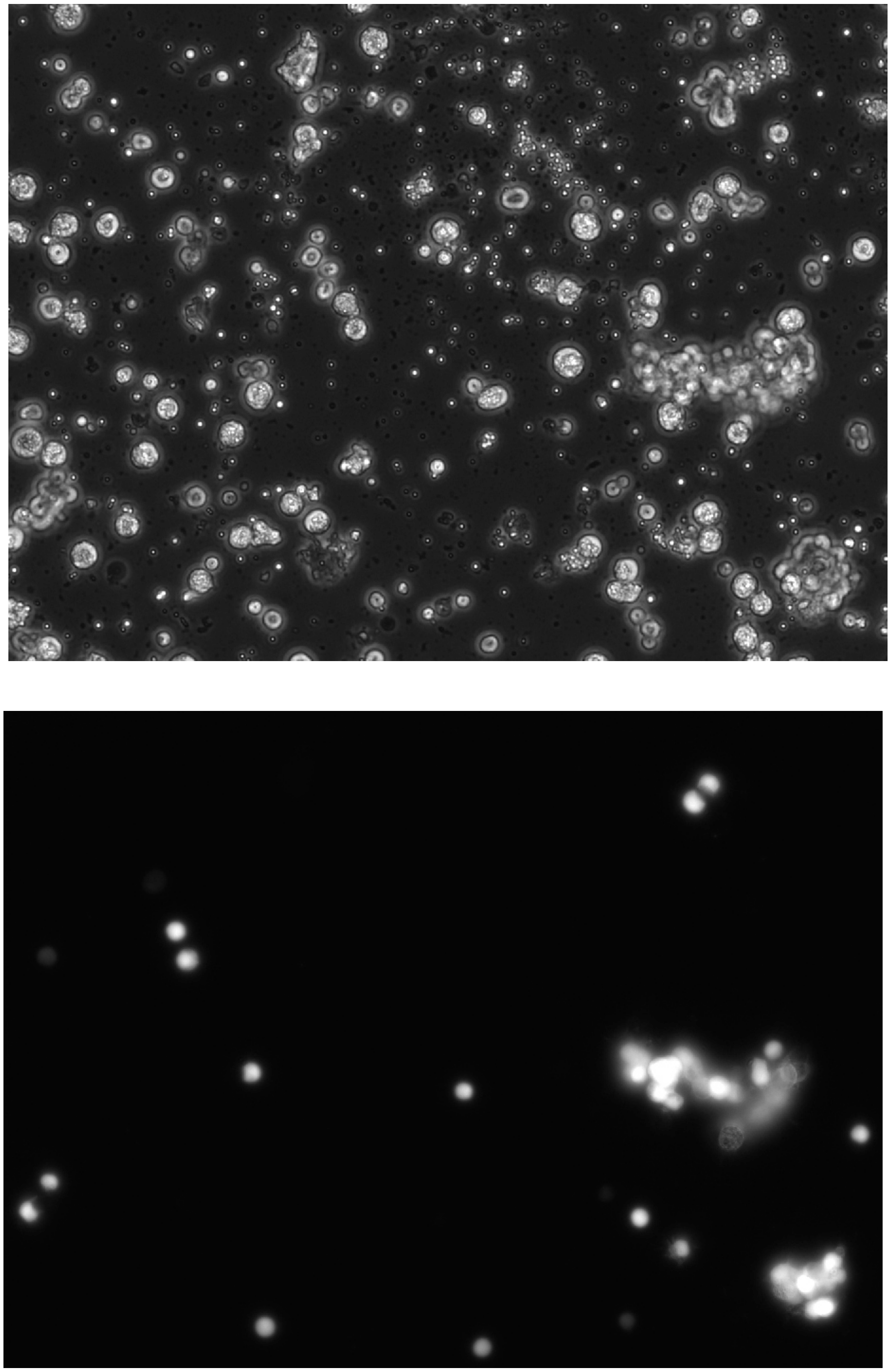

Supplemental Figure 7

D

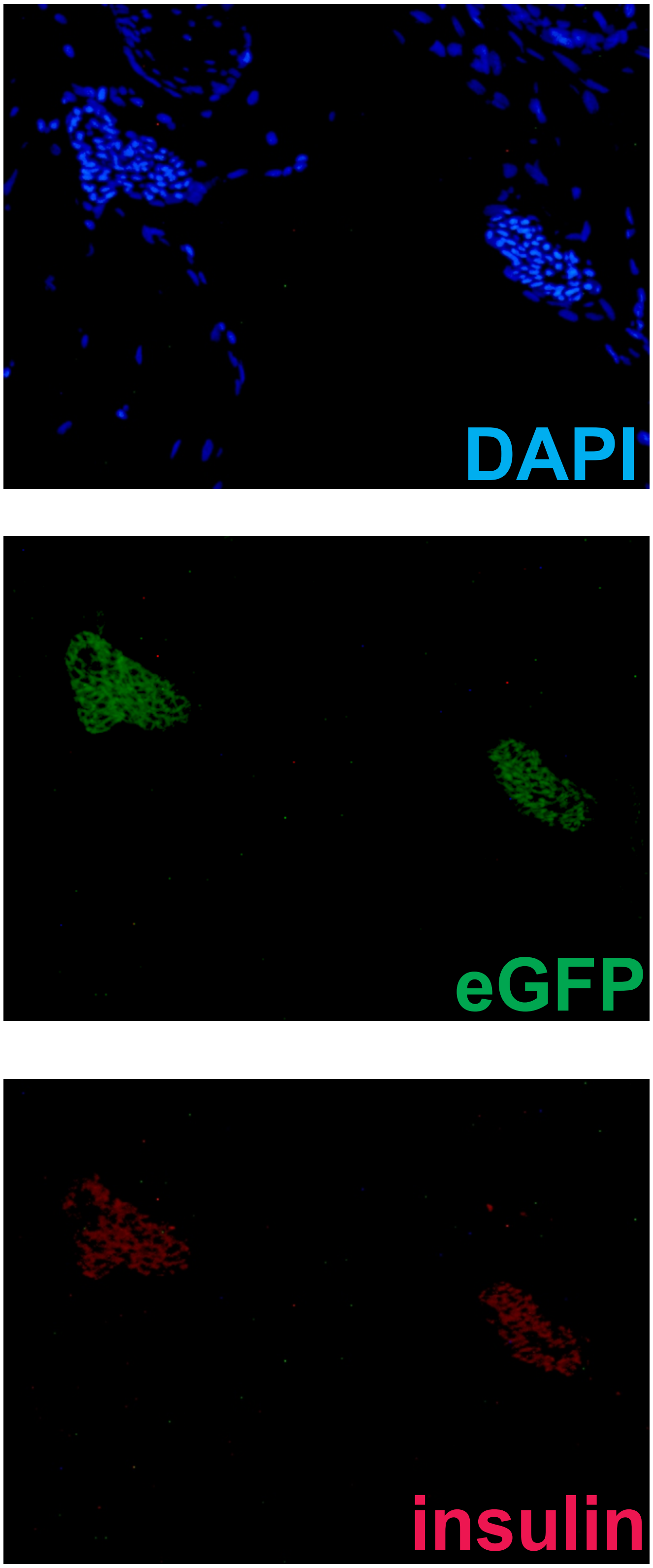

Supplemental Figure 7
