# Supplementary material for: Expression and function of ATP-dependent potassium channels in zebrafish islet β-cells
Source: R Soc Open Sci. 2017 Feb 8;4(2):160808. doi: 10.1098/rsos.160808 (PMC5367309; doi:10.1098/rsos.160808)
Supplement: Supplementary data and original records [file rsos160808supp2.docx]

**Supplemental information concerning**

**Expression and function of ATP-dependent potassium channels in zebrafish islet β-cells**

Christopher H. Emfinger^1,2,3^, Alecia Welscher^2,3^, Zihan Yan^2,3^, Yixi Wang^1,3^, Hannah Conway^1,3^, Jennifer B. Moss^4^, Larry G. Moss^4^, Maria S. Remedi^1,2,3^, and Colin G. Nichols^1,3^

^1^Department of Cell Biology and Physiology, Washington University in St. Louis, St. Louis, MO.

^2^Division of Endocrinology, Metabolism, and Lipid Research; Department of Medicine, Washington University in St. Louis, St. Louis, MO

^3^Center for the Investigation of Membrane Excitability Diseases, Washington University in St. Louis, St. Louis, MO

^4^Division of Endocrinology, Metabolism, and Nutrition and DMPI, Duke University Medical Center, Durham, NC

Address correspondence to Colin G. Nichols ([cnichols@wustl.edu](mailto:cnichols@wustl.edu))

**Condensed title**: “K_ATP_ channels in zebrafish islets”

**Keywords:**

K_ATP_

Metabolism

Pancreas

Zebrafish

Ion Channels

**Supplemental Results**

***Significant residue conservation in SUR1 and Kir6.2 orthologues in zebrafish*** Many residues which, when mutated, cause loss of function in mammalian K_ATP_[1-20], are conserved in zebrafish SUR1 (highlighted green in Supplemental Figure 3; see supplemental ref. for specific residues). Additional residues which, when mutated, have been shown to cause K_ATP_ gain-of-function[21-23] (and consequently diabetes) in mammals, are also conserved in zebrafish SUR1 (also highlighted green in Supplemental Figure 3). Several residues with mutations associated with loss of function[1-3, 12, 24-29] and hyperinsulinemia in mammalian Kir6.2, (Supplemental figure 2, highlighted green), as well as many residues which, when mutated, cause gain of function[30-39] and diabetes are fully conserved (Supplemental figure 2, also highlighted green).

***Not all residues suggested to be functionally significant for mammalian Kir6.2 are conserved in zebrafish Kir6.2 (rel. to Supplemental Fig. 2, highlighted in red)*** The D101 residue in zebrafish Kir6.2 replaces the A101 in human Kir6.2, and the A101D mutation in humans causes congenital hyperinsulinism[1]. L147, found mutated to proline in congenital hyperinsulinism with loss of K_ATP_ channel expression[25], is a valine residue in zebrafish Kir6.2. However, this substitution may be tolerated as both are nonpolar and as valine substitutions for alanine can still form essential tertiary structures in many proteins[40]. S372 and T224, which are phosphorylated by PKA[41, 42], are likewise not conserved. However, it should be noted that it is difficult to assign functional effects to S372 and other nearby residues, as large portions of the C terminal region of zebrafish Kir6.2 differ from those in mammalian Kir6.2.

***Not all residues suggested to be functionally significant for mammalian SUR1 are conserved in zebrafish SUR1 (rel. to Supplemental Fig. 3, highlighted in red)*** Glycine 111 in human SUR1 (which, when mutated to arginine in humans causes congenital hyperinsulinism[3]) is a cysteine in zebrafish SUR1. Valine 187, mutated to aspartic acid in human congenital hyperinsulinism[16], is an isoleucine in zebrafish SUR1. K1336, also linked to hyperinsulinism[43], is a glutamic acid residue in zebrafish SUR1. L1551 in mammalian SUR1[44] is a methionine residue in zebrafish SUR1. Several other residues near the first nucleotide binding fold, mutations at which trigger hyperinsulinism or diabetes in mammals[5, 8, 22, 43], are shifted from those exact positions in zebrafish SUR1 (as is the second walker motif in the first nucleotide binding fold).

**Tables:**

| **Species** | **Component** | **Gene** | **database queried** | **ID query** |
| --- | --- | --- | --- | --- |
| Zebrafish | Sulfonylurea receptor 1 | *ABCC8* | ZFIN | ZDB-TSCRIPT-110325-1796 |
| Human | Sulfonylurea receptor 1 | *ABCC8* | NCBI | 6833 |
| Zebrafish | Sulfonylurea receptor 2 | *ABCC9* | ZFIN | ZDB-TSCRIPT-090929-1334 |
| Human | Sulfonylurea receptor 2, splice isoform A | *ABCC9* | NCBI | NM_005691.3 |
| Human | Sulfonylurea receptor 2, splice isoform B | *ABCC9* | NCBI | NM_020297.3 |
| Human | Potassium voltage-gated channel subfamily J member 11 | *KCNJ11* | NCBI | CCDS31436.1 |
| Human | Potassium voltage-gated channel subfamily J member 8 | *KCNJ8* | NCBI | CCDS8692.1 |
| Zebrafish | Potassium voltage-gated channel subfamily J member 11 | *KCNJ11* | ZFIN | ZDB-TSCRIPT-090929-16640 |
| Zebrafish | Potassium voltage-gated channel subfamily J member 11-like | *KCNJ11L* | ZFIN | ZDB-TSCRIPT-090929-9393 |
| Zebrafish | Potassium voltage-gated channel subfamily J member 8 | *KCNJ8* | ZFIN | ZDB-TSCRIPT-090929-1333 |

**Supplemental table 1:** *References for nucleotide sequence alignments and identity determination.* Databases queried and query IDs for the orthologues of SURx and Kir6.x subunits in humans and zebrafish are indicated.

| **Species** | **Component** | **Gene** | **database queried** | **ID query** |
| --- | --- | --- | --- | --- |
| Zebrafish | Sulfonylurea receptor 1 | *ABCC8* | UniProt | Q5R204 |
| Human | Sulfonylurea receptor 1 | *ABCC8* | UniProt | Q09428 |
| Zebrafish | Sulfonylurea receptor 2 | *ABCC9* | UniProt | Q5RH87 |
| Human | Sulfonylurea receptor 2, splice isoform A | *ABCC9* | UniProt | O60706-1 |
| Human | Sulfonylurea receptor 2, splice isoform B | *ABCC9* | UniProt | O60706-2 |
| Zebrafish | Potassium voltage-gated channel subfamily J member 11 | *KCNJ11* | ZFIN | Q2HX26_DANRE |
| Zebrafish | Potassium voltage-gated channel subfamily J member 8 | *KCNJ11L* | UniProt | Q5R205_DANRE |
| Zebrafish | Potassium voltage-gated channel subfamily J member 8 | *KCNJ8* | ZFIN | Q5RH88_DANRE |

**Supplemental table 2:** *References for amino acid sequence alignments and identity determination.* Databases queried and query IDs for the orthologues of SURx and Kir6.x subunits in humans and zebrafish are indicated.

| **Protein** | **gene** | **database** | **reference** | **primer** | **Sequence** | **Product size (bp)** |
| --- | --- | --- | --- | --- | --- | --- |
| Kir6.3 | *KCNJ11L* | Ensembl | 25:7546242-7552772 | Forward | TGCGGAGCACCAATTGGCCACAAA | 507 (i), 423 (e) |
| Kir6.3 | *KCNJ11L* | Ensembl | 25:7546242-7552772 | Reverse | GCACTTCAATGGAGAACAGAAAAG | 507 (i), 423 (e) |
| Kir6.1 | *KCNJ8* | Ensembl | 4:14718543-14721247 | Forward | GCACGGAGATCTGGACACC | 1248 (i), 146 (e) |
| Kir6.1 | *KCNJ8* | Ensembl | 4:14718543-14721247 | Reverse | TGGGACACCGCTCTGTTATCATAC | 1248 (i), 146 (e) |
| Kir6.2 | *KCNJ11* | NCBI | NM_001039827.1 | Forward | GCGAACAGGGACGGTTTCTA | 97 |
| Kir6.2 | *KCNJ11* | NCBI | NM_001039827.1 | Reverse | CTGCACAGGAAGGACATGGT | 97 |
| SUR1 | *ABCC8* | Ensembl | 25:22709793-22710343 | Forward | CGGACATTGACATCCTGCCTCA | 551 (i), 128 (e) |
| SUR1 | *ABCC8* | Ensembl | 25:22709793-22710343 | Reverse | GAACACCACATTGGTAGTTTG | 551 (i), 128 (e) |
| SUR2 | *ABCC9* | NCBI | NM_001030154.1 | Forward | TGCGTCTCTCCACTTCCAAC | 190 |
| SUR2 | *ABCC9* | NCBI | NM_001030154.1 | Reverse | CCTATCAATGCGCTGTTGCC | 190 |
| SUR2 | *ABCC9* | Ensembl | 4:14693035-14696159 | Forward | GATGACCCTTTTTCTGCCTTGGA | 401 (i), 257 (e) |
| SUR2 | *ABCC9* | Ensembl | 4:14693035-14696159 | Reverse | AGCTCTTGATCTTGTCTGTTC | 401 (i), 257 (e) |

**Supplemental table 3:** *Sequences and relevant data for the primers used in PCR reactions for the K_ATP_ channel subunits in zebrafish*. For primers with products differing by whether the sequence contains an intron, the product length for samples containing the targed (i) precedes the length of the target which does not contain introns (e). For figure 3 A, *ABCC9* primer set from the NCBI reference was used. For Figure 3 B, the *ABCC9* primer set for the Ensembl sequence reference was used. References for Ensembl are Chromosome #: nucleotide position. The reference genome for Ensembl queries was GRCz10.

**Supplemental figure legends**

Supplemental Figure 1: *Zebrafish possess orthologues for K_ATP_ channels with high amino acid conservation*. At low plasma glucose (A, left), mammalian β-cell K_ATP_ channels are open due to low intracellular [ATP]/[ADP], hyperpolarizing the cell. As plasma glucose rises (A, right), glucose enters the cell through Glut2 and is metabolized to ATP, increasing [ATP]/[ADP]. This inhibits K_ATP_ channels, and V_m_ increases, opening VDCCs. Influx of calcium triggers insulin secretion. Orthologues of many components of mammalian metabolism-secretion coupling are present in the zebrafish, including K_ATP_ channels (single channel diagram in B, top view). Identity alignments for amino acid sequences for Kir6.x and SURx subunits (B) are scaled, with darker grey indicating more identity between the proteins compared. The query IDs for amino acid alignments are indicated in Supplemental table 2. There is also high identity between the nucleotide sequences for these genes (C). Query IDs for nucleotide alignments are in supplemental table 1.

Supplemental Figure 2: *Residues suggested to be functionally significant for mammalian Kir6.2 are conserved in zebrafish Kir6.2* Alignments for the Kir6.x subunits from human and zebrafish are shown highlighting residues suggested to be significant for gain-of-function (neonatal diabetes) or loss-of-function (congenital hyperinsulinism). Green highlighting indicates conservation of the residue; red highlighting indicates the residue is not conserved. Red box outlines indicate regulatory residues post-translationally modified in mammalian Kir6.2 which are not conserved in zebrafish Kir6.2, while green outline boxes show residues which are post-translationally modified in mammalian Kir6.2 that are conserved in zebrafish Kir6.2.

Supplemental Figure 3: *Many residues with functional significance in mammalian SUR1 are conserved in zebrafish SUR1.* The sequences for zebrafish SUR1, zebrafish SUR2, and human SUR2A and SUR2B are shown aligned to human SUR1. Residues identical to those in human SUR1 are highlighted in grey. Specific residues of functional significance to human SUR1 which are conserved in zebrafish SUR1 are highlighted green. Green outlines highlight conserved residues which are important for subunit regulation in mammals. The predicted nucleotide binding folds are indicated by blue outlines. Within these, red outlines highlight the Walker motifs. Residues highlighted red are significant for SUR1 function in mammals, but are not identical in zebrafish SUR1. Above the residue ruler, two bars indicate the topological domains predicted by Interpro 5 software for zebrafish (upper bar) and human (lower bar) SUR1. Pink color (light for zebrafish, dark for human) indicates predicted cytoplasmic domains. Blue color (light for zebrafish, dark for human) indicates predicted extracellular domains. Grey color (zebrafish) and black color (human) indicate predicted transmembrane domains. Query IDs for these sequences are shown in supplemental table 2.

Supplemental Figure 4: *Original images for PCR results in Figure 3*. The original images for the PCR results for Figure 3 (left) are shown here for (A) *KCNJ11*, (B) *ABCC8*, (C) *KCNJ8*, (D) *ABCC9*, and (E) *KCNJ11L*. “cDNA” denotes complementary DNA generated from isolated RNA. “gDNA” denotes genomic DNA. RNA denotes sample used to prepare given cDNA without reverse transcriptase. Plasmid is a pKSII/INSeGFP construct (Addgene 53787). Certain images in which the ladder is faint have additionally notated contrast-enhanced images presented to their right to show the final bands of the ladder.

Supplemental Figure 5: *Original images for PCR results in Figure 3*. The original images for the PCR results for Figure 3 (right) are shown here for (A) *KCNJ11*, (B) *ABCC8*, (C) *KCNJ8*, (D) *ABCC9*, and (E) *KCNJ11L*. “cDNA” denotes complementary DNA generated from isolated RNA. “gDNA” denotes genomic DNA. RNA denotes sample used to prepare given cDNA without reverse transcriptase. Plasmid is a pKSII/INSeGFP construct (Addgene 53787). Certain images in which the ladder is faint have additionally notated contrast-enhanced images presented to their right to show the final bands of the ladder.

Supplemental figure 6: *Complete example traces which were shown in Figure 4 for tolbutamide and glibenclamide.*

Supplemental figure 7: *Original microscopy images in Figure 1.* (A) Bright field (top) and green fluorescence (bottom) of an adult (12 week old) zebrafish anesthetized on its left side (10x). These images were combined and rotated prior to their use in Figure 1, as indicated in Figure 1 legend. Bright-field image was contrast-enhanced prior to being super-imposed with fluorescent image to improve visibility within final combined image. (B) Bright field (top) and green fluorescence (bottom) images of isolated zebrafish islets (20x). (C) Bright field (top) and green fluorescence (bottom) images of dispersed zebrafish β-cells (40x). (D) Co-localization of insulin and eGFP staining in zebrafish histological sections confirms specificity of insulin promoter driving eGFP (20x).

**Supplemental Bibliography**

[1] Suchi, M., MacMullen, C.M., Thornton, P.S., Adzick, N.S., Ganguly, A., Ruchelli, E.D. & Stanley, C.A. 2005 Molecular and immunohistochemical analyses of the focal form of congenital hyperinsulinism. *Mod Pathol* **19**, 122-129.

[2] Henwood, M.J., Kelly, A., MacMullen, C., Bhatia, P., Ganguly, A., Thornton, P.S. & Stanley, C.A. 2005 Genotype-Phenotype Correlations in Children with Congenital Hyperinsulinism Due to Recessive Mutations of the Adenosine Triphosphate-Sensitive Potassium Channel Genes. *The Journal of Clinical Endocrinology & Metabolism* **90**, 789-794. (doi:10.1210/jc.2004-1604).

[3] Tornovsky, S., Crane, A., Cosgrove, K.E., Hussain, K., Lavie, J., Heyman, M.a., Nesher, Y., Kuchinski, N.a., Ben-Shushan, E., Shatz, O., et al. 2004 Hyperinsulinism of Infancy: Novel ABCC8 and KCNJ11 Mutations and Evidence for Additional Locus Heterogeneity. *The Journal of Clinical Endocrinology & Metabolism* **89**, 6224-6234. (doi:10.1210/jc.2004-1233).

[4] Nestorowicz, A., Glaser, B., Wilson, B.A., Shyng, S.-L., Nichols, C.G., Stanley, C.A., Thornton, P.S. & Permutt, M.A. 1998 Genetic Heterogeneity in Familial Hyperinsulinism. *Human Molecular Genetics* **7**, 1119-1128. (doi:10.1093/hmg/7.7.1119).

[5] Fernández–Marmiesse, A., Salas, A., Vega, A., Fernández–Lorenzo, J.R., Barreiro, J. & Carracedo, Á. 2006 Mutation spectra of ABCC8 gene in Spanish patients with hyperinsulinism of infancy (HI). *Human Mutation* **27**, 214-214. (doi:10.1002/humu.9401).

[6] Yan, F.-F., Lin, Y.-W., MacMullen, C., Ganguly, A., Stanley, C.A. & Shyng, S.-L. 2007 Congenital Hyperinsulinism–Associated ABCC8 Mutations That Cause Defective Trafficking of ATP-Sensitive K(+) Channels: Identification and Rescue. *Diabetes* **56**, 2339-2348. (doi:10.2337/db07-0150).

[7] Babenko, A.P., Polak, M., Cavé, H., Busiah, K., Czernichow, P., Scharfmann, R., Bryan, J., Aguilar-Bryan, L., Vaxillaire, M. & Froguel, P. 2006 Activating Mutations in the ABCC8 Gene in Neonatal Diabetes Mellitus. *New England Journal of Medicine* **355**, 456-466. (doi:10.1056/NEJMoa055068).

[8] Saint-Martin, C., Zhou, Q., Martin, G.M., Vaury, C., Leroy, G., Arnoux, J.-B., de Lonlay, P., Shyng, S.-L. & Bellanné-Chantelot, C. 2015 Monoallelic ABCC8 mutations are a common cause of diazoxide-unresponsive diffuse form of congenital hyperinsulinism. *Clinical genetics* **87**, 448-454. (doi:10.1111/cge.12428).

[9] Shyng, S.L., Ferrigni, T., Shepard, J.B., Nestorowicz, A., Glaser, B., Permutt, M.A. & Nichols, C.G. 1998 Functional analyses of novel mutations in the sulfonylurea receptor 1 associated with persistent hyperinsulinemic hypoglycemia of infancy. *Diabetes* **47**, 1145-1151. (doi:10.2337/diabetes.47.7.1145).

[10] Magge, S.N., Shyng, S.-L., MacMullen, C., Steinkrauss, L., Ganguly, A., Katz, L.E.L. & Stanley, C.A. 2004 Familial Leucine-Sensitive Hypoglycemia of Infancy Due to a Dominant Mutation of the β-Cell Sulfonylurea Receptor. *The Journal of Clinical Endocrinology & Metabolism* **89**, 4450-4456. (doi:10.1210/jc.2004-0441).

[11] Nestorowicz, A., Wilson, B.A., Schoor, K.P., Inoue, H., Glaser, B., Landau, H., Stanley, C.A., Thornton, P.S., Clement, J.P., Bryan, J., et al. 1996 Mutations in the Sulfonylurea Receptor Gene Are Associated with Familial Hyperinsulinism in Ashkenazi Jews. *Human Molecular Genetics* **5**, 1813-1822. (doi:10.1093/hmg/5.11.1813).

[12] Ohkubo, K., Nagashima, M., Naito, Y., Taguchi, T., Suita, S., Okamoto, N., Fujinaga, H., Tsumura, K., Kikuchi, K. & Ono, J. 2005 Genotypes of the pancreatic β-cell K-ATP channel and clinical phenotypes of Japanese patients with persistent hyperinsulinaemic hypoglycaemia of infancy. *Clinical Endocrinology* **62**, 458-465. (doi:10.1111/j.1365-2265.2005.02242.x).

[13] Thornton, P.S., MacMullen, C., Ganguly, A., Ruchelli, E., Steinkrauss, L., Crane, A., Aguilar-Bryan, L. & Stanley, C.A. 2003 Clinical and Molecular Characterization of a Dominant Form of Congenital Hyperinsulinism Caused by a Mutation in the High-Affinity Sulfonylurea Receptor. *Diabetes* **52**, 2403-2410. (doi:10.2337/diabetes.52.9.2403).

[14] de Lonlay-Debeney, P., Poggi-Travert, F., Fournet, J.-C., Sempoux, C., Vici, C.D., Brunelle, F., Touati, G., Rahier, J., Junien, C., Nihoul-Fékété, C., et al. 1999 Clinical Features of 52 Neonates with Hyperinsulinism. *New England Journal of Medicine* **340**, 1169-1175. (doi:10.1056/NEJM199904153401505).

[15] Tanizawa, Y., Matsuda, K., Matsuo, M., Ohta, Y., Ochi, N., Adachi, M., Koga, M., Mizuno, S., Kajita, M., Tanaka, Y., et al. 2000 Genetic analysis of Japanese patients with persistent hyperinsulinemic hypoglycemia of infancy: nucleotide-binding fold-2 mutation impairs cooperative binding of adenine nucleotides to sulfonylurea receptor 1. *Diabetes* **49**, 114-120. (doi:10.2337/diabetes.49.1.114).

[16] Huopio, H., Jääskeläinen, J., Komulainen, J., Miettinen, R., Kärkkäinen, P., Laakso, M., Tapanainen, P., Voutilainen, R. & Otonkoski, T. 2002 Acute Insulin Response Tests for the Differential Diagnosis of Congenital Hyperinsulinism. *The Journal of Clinical Endocrinology & Metabolism* **87**, 4502-4507. (doi:10.1210/jc.2002-020378).

[17] Nichols, C.G., Shyng, S.-L., Nestorowicz, A., Glaser, B., Clement, J.P., Gonzalez, G., Aguilar-Bryan, L., Permutt, M.A. & Bryan, J. 1996 Adenosine Diphosphate as an Intracellular Regulator of Insulin Secretion. *Science* **272**, 1785-1787. (doi:10.1126/science.272.5269.1785).

[18] Taschenberger, G., Mougey, A., Shen, S., Lester, L.B., LaFranchi, S. & Shyng, S.-L. 2002 Identification of a Familial Hyperinsulinism-causing Mutation in the Sulfonylurea Receptor 1 That Prevents Normal Trafficking and Function of KATP Channels. *Journal of Biological Chemistry* **277**, 17139-17146.

[19] Otonkoski, T., Näntö-Salonen, K., Seppänen, M., Veijola, R., Huopio, H., Hussain, K., Tapanainen, P., Eskola, O., Parkkola, R., Ekström, K., et al. 2006 Noninvasive Diagnosis of Focal Hyperinsulinism of Infancy With [18F]-DOPA Positron Emission Tomography. *Diabetes* **55**, 13-18. (doi:10.2337/diabetes.55.01.06.db05-1128).

[20] Shepherd, R., Cosgrove, K., O'Brien, R., Barnes, P., Ammala, C. & Dunne, M. 2000 Hyperinsulinism of infancy: towards an understanding of unregulated insulin release. *Archives of Disease in Childhood. Fetal and Neonatal Edition* **82**, F87-F97. (doi:10.1136/fn.82.2.F87).

[21] Ellard, S., Flanagan, Sarah E., Girard, Christophe A., Patch, A.-M., Harries, Lorna W., Parrish, A., Edghill, Emma L., Mackay, Deborah J G., Proks, P., Shimomura, K., et al. 2007 Permanent Neonatal Diabetes Caused by Dominant, Recessive, or Compound Heterozygous SUR1 Mutations with Opposite Functional Effects. *American Journal of Human Genetics* **81**, 375-382.

[22] Vaxillaire, M., Dechaume, A., Busiah, K., Cavé, H., Pereira, S., Scharfmann, R., de Nanclares, G.P., Castano, L., Froguel, P. & Polak, M. 2007 New ABCC8 Mutations in Relapsing Neonatal Diabetes and Clinical Features. *Diabetes* **56**, 1737-1741. (doi:10.2337/db06-1540).

[23] Küçükemre Ay, B., Bundak, R., Baş, F., Maraş, H., Saka, N., Günöz, H. & Darendeliler, F. 2012 Permanent Neonatal Diabetes Mellitus: Same Mutation, Different Glycemic Control with Sulfonylurea Therapy on Long-Term Follow-up. *Journal of Clinical Research in Pediatric Endocrinology* **4**, 107-110. (doi:10.4274/Jcrpe.524).

[24] Lin, Y.-W., MacMullen, C., Ganguly, A., Stanley, C.A. & Shyng, S.-L. 2006 A NOVEL KCNJ11 MUTATION ASSOCIATED WITH CONGENITAL HYPERINSULINISM REDUCES THE INTRINSIC OPEN PROBABILITY OF β-CELL ATP-SENSITIVE POTASSIUM CHANNELS. *The Journal of biological chemistry* **281**, 3006-3012. (doi:10.1074/jbc.M511875200).

[25] Aguilar-Bryan, L. & Bryan, J. 1999 Molecular Biology of Adenosine Triphosphate-Sensitive Potassium Channels. *Endocrine Reviews* **20**, 101-135. (doi:10.1210/edrv.20.2.0361).

[26] Pinney, S.E., MacMullen, C., Becker, S., Lin, Y.-W., Hanna, C., Thornton, P., Ganguly, A., Shyng, S.-L. & Stanley, C.A. 2008 Clinical characteristics and biochemical mechanisms of congenital hyperinsulinism associated with dominant K(ATP )channel mutations. *The Journal of Clinical Investigation* **118**, 2877-2886. (doi:10.1172/JCI35414).

[27] Marthinet, E., Bloc, A., Oka, Y., Tanizawa, Y., Wehrle-Haller, B., Bancila, V., Dubuis, J.-M., Philippe, J. & Schwitzgebel, V.M. 2005 Severe Congenital Hyperinsulinism Caused by a Mutation in the Kir6.2 Subunit of the Adenosine Triphosphate-Sensitive Potassium Channel Impairing Trafficking and Function. *The Journal of Clinical Endocrinology & Metabolism* **90**, 5401-5406. (doi:10.1210/jc.2005-0202).

[28] Taneja, T.K., Mankouri, J., Karnik, R., Kannan, S., Smith, A.J., Munsey, T., Christesen, H.B.T., Beech, D.J. & Sivaprasadarao, A. 2009 Sar1-GTPase-dependent ER exit of KATP channels revealed by a mutation causing congenital hyperinsulinism. *Human Molecular Genetics* **18**, 2400-2413. (doi:10.1093/hmg/ddp179).

[29] Mannikko, R., Flanagan, S.E., Sim, X., Segal, D., Hussain, K., Ellard, S., Hattersley, A.T. & Ashcroft, F.M. 2011 Mutations of the same conserved glutamate residue in NBD2 of the sulfonylurea receptor 1 subunit of the KATP channel can result in either hyperinsulinism or neonatal diabetes. *Diabetes* **60**, 1813-1822. (doi:10.2337/db10-1583).

[30] Vaxillaire, M., Populaire, C., Busiah, K., Cavé, H., Gloyn, A.L., Hattersley, A.T., Czernichow, P., Froguel, P. & Polak, M. 2004 Kir6.2 Mutations Are a Common Cause of Permanent Neonatal Diabetes in a Large Cohort of French Patients. *Diabetes* **53**, 2719-2722. (doi:10.2337/diabetes.53.10.2719).

[31] Flanagan, S.E., Edghill, E.L., Gloyn, A.L., Ellard, S. & Hattersley, A.T. 2006 Mutations in KCNJ11, which encodes Kir6.2, are a common cause of diabetes diagnosed in the first 6 months of life, with the phenotype determined by genotype. *Diabetologia* **49**, 1190-1197. (doi:10.1007/s00125-006-0246-z).

[32] Shimomura, K., Girard, C.A.J., Proks, P., Nazim, J., Lippiat, J.D., Cerutti, F., Lorini, R., Ellard, S., Hattersley, A.T., Barbetti, F., et al. 2006 Mutations at the Same Residue (R50) of Kir6.2 (KCNJ11) That Cause Neonatal Diabetes Produce Different Functional Effects. *Diabetes* **55**, 1705-1712. (doi:10.2337/db05-1640).

[33] Männikkö, R., Jefferies, C., Flanagan, S.E., Hattersley, A., Ellard, S. & Ashcroft, F.M. 2010 Interaction between mutations in the slide helix of Kir6.2 associated with neonatal diabetes and neurological symptoms. *Human Molecular Genetics* **19**, 963-972. (doi:10.1093/hmg/ddp554).

[34] Shimomura, K., Hörster, F., de Wet, H., Flanagan, S.E., Ellard, S., Hattersley, A.T., Wolf, N.I., Ashcroft, F. & Ebinger, F. 2007 A novel mutation causing DEND syndrome: A treatable channelopathy of pancreas and brain. *Neurology* **69**, 1342-1349. (doi:10.1212/01.wnl.0000268488.51776.53).

[35] Tammaro, P. & Ashcroft, F.M. 2007 A mutation in the ATP-binding site of the Kir6.2 subunit of the K(ATP) channel alters coupling with the SUR2A subunit. *The Journal of Physiology* **584**, 743-753. (doi:10.1113/jphysiol.2007.143149).

[36] Yorifuji, T., Nagashima, K., Kurokawa, K., Kawai, M., Oishi, M., Akazawa, Y., Hosokawa, M., Yamada, Y., Inagaki, N. & Nakahata, T. 2005 The C42R Mutation in the Kir6.2 (KCNJ11) Gene as a Cause of Transient Neonatal Diabetes, Childhood Diabetes, or Later-Onset, Apparently Type 2 Diabetes Mellitus. *The Journal of Clinical Endocrinology & Metabolism* **90**, 3174-3178. (doi:10.1210/jc.2005-0096).

[37] Gloyn, A.L., Reimann, F., Girard, C., Edghill, E.L., Proks, P., Pearson, E.R., Temple, I.K., Mackay, D.J.G., Shield, J.P.H., Freedenberg, D., et al. 2005 Relapsing diabetes can result from moderately activating mutations in KCNJ11. *Human Molecular Genetics* **14**, 925-934. (doi:10.1093/hmg/ddi086).

[38] Bonnefond, A., Philippe, J., Durand, E., Dechaume, A., Huyvaert, M., Montagne, L., Marre, M., Balkau, B., Fajardy, I., Vambergue, A., et al. 2012 Whole-Exome Sequencing and High Throughput Genotyping Identified KCNJ11 as the Thirteenth MODY Gene. *PLoS ONE* **7**, e37423. (doi:10.1371/journal.pone.0037423).

[39] Florez, J.C., Jablonski, K.A., Kahn, S.E., Franks, P.W., Dabelea, D., Hamman, R.F., Knowler, W.C., Nathan, D.M. & Altshuler, D. 2007 Type 2 Diabetes–Associated Missense Polymorphisms KCNJ11 E23K and ABCC8 A1369S Influence Progression to Diabetes and Response to Interventions in the Diabetes Prevention Program. *Diabetes* **56**, 531-536. (doi:10.2337/db06-0966).

[40] Gregoret, L.M. & Sauer, R.T. 1998 Tolerance of a protein helix to multiple alanine and valine substitutions. *Folding and Design* **3**, 119-126. (doi:<http://dx.doi.org/10.1016/S1359-0278(98)00017-0>).

[41] Aziz, Q., Thomas, A.M., Khambra, T. & Tinker, A. 2011 The regulation of the KATP channel subunit, KIR6.2, by a CA2+-dependent protein kinase C. *Journal of Biological Chemistry*. (doi:10.1074/jbc.M111.243923).

[42] Lin, Y.-F., Jan, Y.N. & Jan, L.Y. 2000 Regulation of ATP-sensitive potassium channel function by protein kinase A-mediated phosphorylation in transfected HEK293 cells. *The EMBO Journal* **19**, 942-955. (doi:10.1093/emboj/19.5.942).

[43] Snider, K.E., Becker, S., Boyajian, L., Shyng, S.L., MacMullen, C., Hughes, N., Ganapathy, K., Bhatti, T., Stanley, C.A. & Ganguly, A. 2013 Genotype and Phenotype Correlations in 417 Children With Congenital Hyperinsulinism. *The Journal of Clinical Endocrinology and Metabolism* **98**, E355-E363. (doi:10.1210/jc.2012-2169).

[44] Campbell, J.D., Sansom, M.S.P. & Ashcroft, F.M. 2003 Potassium channel regulation. *EMBO Reports* **4**, 1038-1042. (doi:10.1038/sj.embor.7400003).
